# Scrambled RGD Hexameric Peptide Hydrogel Supports Efficient Self‐Assembly and Cell Activity

**DOI:** 10.1002/chem.202404410

**Published:** 2025-04-21

**Authors:** Karrar Al Taief, Stephanie Nemec, Isis A. Middleton, Kristopher A. Kilian, Pall Thordarson

**Affiliations:** ^1^ School of Chemistry University of New South Wales Sydney NSW 2052 Australia; ^2^ The UNSW RNA Institute University of New South Wales Sydney NSW 2052 Australia; ^3^ School of Materials Science and Engineering University of New South Wales Sydney NSW 2052 Australia; ^4^ Australian Centre for NanoMedicine University of New South Wales Sydney NSW 2052 Australia

**Keywords:** biomaterials, hydrogels, rgd, rheology, self‐assembled peptides

## Abstract

The amino acid sequence is crucial in controlling peptide‐based hydrogel formation, whereby changing the position of a single amino acid can significantly alter the gel's properties. Herein, we report the gelation kinetics and cell viability of scrFmoc‐GFFRDG (where we have scrambled the RGD‐based gel hexapeptide; Fmoc‐GFFRGD). The scrambled sequence showed improved gelation properties compared to the original Fmoc‐GFFRGD sequence, with scrFmoc‐GFFRDG forming a gel in under 10 min, significantly faster than the 2‐h gelation time, and at a concentration eight times lower than the original Fmoc‐GFFRGD sequence. We also examined the combination of the two gelators in a ratio of 1:1, final concentration of 0.4% (w/v). Interestingly, the stiffness of the hybrid hydrogel was ∼3 kPa, whereas individually, neither gelator at the same concentration exceeded 0.5 kPa. The cell‐adhesion motif RGD improves the ability of the peptides to promote attachment of cells due to integrin recognition. However, when fibroblasts were cultured on the hydrogels, scrFmoc‐GFFRDG yielded a higher level of α‐SMA expression in cells than those cultured on Fmoc‐GFFRGD, suggesting a microenvironment conducive to myofibroblast transitions. This study provides a new outlook on how a well‐known scrambled peptide motif (RDG) can fine‐tune hydrogel assembly and cell culture applications.

## Graphical abstract

Self‐assembled peptide hydrogels that incorporate bioactive motifs such as the integrin‐binding epitope Arginine‐Glycine‐Aspartic acid (RGD), are known for supporting cell growth. Surprisingly, scrambling the peptide cell‐adhesion motif RGD to RDG in an Fmoc‐hexapeptide peptide gelator (Fmoc‐GFFRGD) results in superior properties in terms of minimum gelation concentration and biocompatibility.

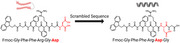

## 1 Introduction

Peptides often possess intrinsic structures resulting from the composition and order of the amino acids. Capping the N‐terminus of peptides can further alter their structure and trigger self‐assembly into fibers.^[^ ^1^ ^]^ When these fibers assemble into networks, they can trap water molecules, forming hydrogels.^[^ ^2^ ^]^ Peptide hydrogels have a range of biomedical applications, including drug delivery and tissue engineering.^[^ ^3^ ^]^

Altering the order of amino acids in a peptide can drastically change how the molecules self‐assemble, due to intramolecular hydrogen bonding, hydrophobic interactions between adjacent aromatic moieties, and changes in charge distribution.^[^ ^4^ ^]^ Libraries of peptide hydrogels with different structural properties have been generated by manipulating the sequence of amino acids.^[^ ^5^ ^]^ Due to their tunable properties, there has been growing interest in using peptide hydrogels as 3D cell culture environments.^[^ ^6^ ^]^

When considering the design of peptides for hydrogels capable of 3D cell culture, they must exhibit strong gelation properties and include biocompatible motifs that support cell adhesion, spreading, and migration via integrins.^[^ ^7^ ^]^ Typically, these are short motifs like Arginine‐Glycine‐Aspartic acid (RGD),^[^ ^8^ ^]^ Isoleucine‐Lysine‐Valine‐Alanine‐Valine(IKVAV),^[^ ^9^ ^]^ and Glycine‐Glutamic acid‐Arginine (GER),^[^ ^10^ ^]^ derived from naturally occurring extracellular matrix proteins: fibronectin, laminin, and collagen, respectively. In vitro, these proteins are mimicked by selectively synthesizing the bio‐functional epitope to which cell integrins adhere.

The RGD peptide is a conserved motif in many extracellular matrix proteins^[^ ^11^ ^]^ and is recognized by the cell surface integrins, a family of transmembrane receptors involved in “feeling” the extracellular environment. Integrin recognition of RGD and other adhesion sequences is an important step in ensuring cells can sense their environment, which has important functions in supporting cell migration, proliferation, and differentiation. Interestingly, there have also been examples of scrambled versions of the binding motif displaying good cell compatibility. Substitutions of the glycine amino acid with valine and alanine leave the cell adhesion properties of the matrix unaffected.^[^ ^12^ ^]^ For example, the oligopeptide, RAD16, which features two substitutions of glycine to alanine, was assembled into a macroscopic membranous matrix capable of supporting 14 different cell types.^[^ ^13^ ^]^ In another study, RDG—a scrambled negative control of the RGD motif from fibronectin—was incorporated as a building block into a recombinant protein, showing no toxicity towards Schwann cells within the measured time frame.^[^ ^14^ ^]^ They hypothesize that, over a longer time frame, the lack of a binding motif within the extracellular matrix may trigger anoikis. Moreover, when scrambling the sequence RGD to GRD, the tripeptide was unable to support and bind bovine fibroblasts. These studies indicate the difficulty in predicting the biocompatibility of peptides purely based on the sequence.

The change in cell compatibility of the peptide hydrogel that occurs when altering the amino acid sequence could be a result of the altered self‐assembly.^[^ ^4^, ^15^ ^]^ Three pentapeptide hydrogels were formed from different sequences of isoleucine (I) and aspartic acid (D), wherein, at 1% (w/v), DIIID had 10‐times the storage modulus of IDIDI.^[^ ^4a^ ^]^ The mechanical properties of hydrogels were significantly influenced by the charge distribution from the aspartic acids, altering the ability of peptides to form intramolecular salt bridges. Salt bridges are often observed in peptide sequences with positive charges from lysine (Lys: K^+^) and arginine (Arg: R+) and negative charges from aspartic acid (Asp: D−) and glutamic acid (Glu: E−).^[^ ^16^ ^]^

Previously, we reported a peptide hydrogel Fmoc‐GFFRGD^[^ ^17^ ^]^ which was inspired by the incorporation of the fibronectin motif RGD into a well‐known peptide gelator, Fmoc‐GFF.^[^ ^1^ ^]^ In this work, the peptide sequence, Fmoc‐GFFRGD, was scrambled, switching the positions of the charged amino acids (R+) and (D−), to explore how this would affect the intermolecular interactions of the resulting Fmoc‐peptide. In short, the two differ by the fact that in RGD, the neutral glycine separates the two charged amino acids (R+) and (D−) with an additional negative charge on D as it is the C‐terminus of our peptide, whereas in our scrambled RDG, the two charged amino acids (R+) and (D−) are next to one another and the terminal neutral glycine (G) carries the C‐terminus charge under physiological conditions. Herein, we report improved gelation kinetics of the scrambled sequence, scrFmoc‐GFFRDG, compared to the original, Fmoc‐GFFRGD. Three hydrogels are assembled and compared in detail in this study: the parent Fmoc‐GFFRGD, the scrambled scrFmoc‐GFFRDG, and a hybrid formed from a mixture of the two. The structural properties of the resulting hydrogels were characterized by spectroscopy and microscopy, and their biocompatibility was investigated using MRC‐5 fibroblast and cancer‐associated fibroblast.

## 2 Results and Discussion

Peptides were synthesized by standard solid‐phase peptide synthesis methods and characterized using NMR, HPLC, and mass spectrometry (see supporting information, S2, for full details). Hydrogel self‐assembly was triggered by dissolving the peptides in 0.1 M sodium hydroxide and adding Dulbecco's Modified Eagle Medium (DMEM) at 37 °C, resulting in a gel that is in water:DMEM (1:1), as we have previously described.^[^ ^17^ ^]^

### 2.1 Gel Characterization

**Figure 1:**
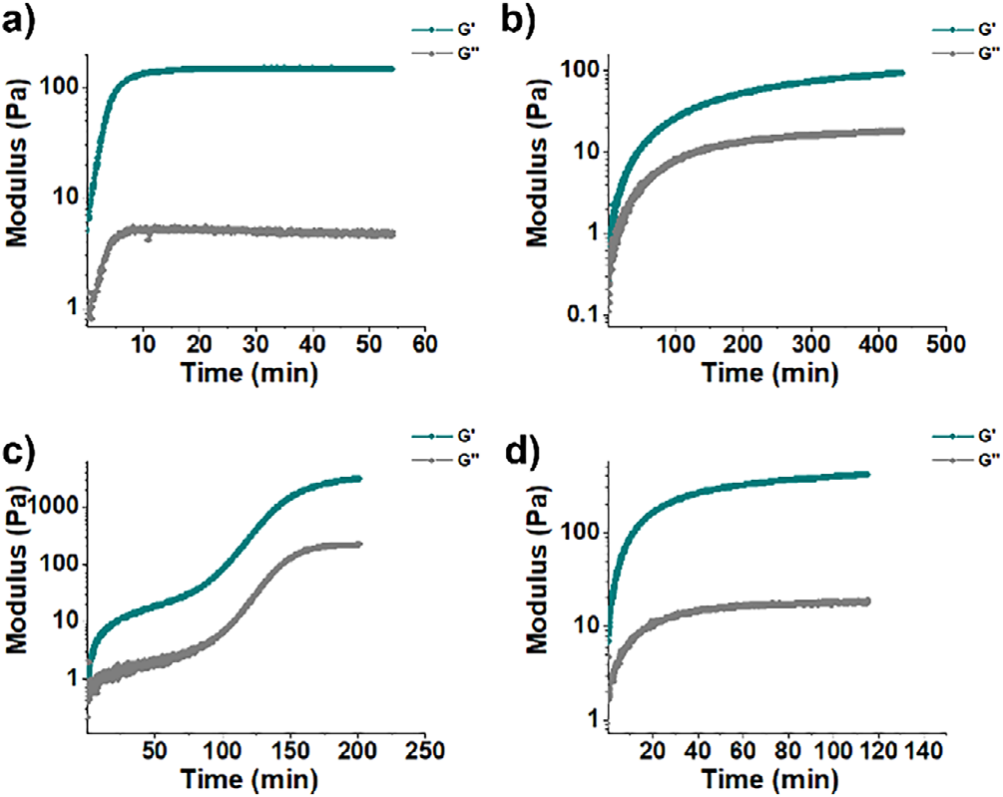
Rheological time sweep measurements at 37 °C in water:DMEM (1:1, v/v, DMEM = Dulbecco's Modified Eagle Medium) of a) 0.05% (w/v) scrFmoc‐GFFRDG, b) 0.4% (w/v) of Fmoc‐GFFRGD, c) 1:1 ratio 0.4% (w/v) of Fmoc‐GFFRGD/scrFmoc‐GFFRDG (0.2%/0.2%, w/w/v, of each) and d) 0.4% (w/v) of scrFmoc‐GFFRDG. Both the storage (G’) and loss (G’’) modulus as a function of time are shown.

The gelation kinetics of five gels in water:DMEM (1:1) at 37 °C were investigated, corresponding to each of the peptides at low concentration (0.05%, w/v), high concentration (0.40%, w/v), and a hybrid of the two combined (0.20%/0.20%, w/w/v) (Figure 1 and Table 1). We established that Fmoc‐GFFRGD has a minimum gelation concentration of 0.4% (w/v), which was a little lower than previously reported,^[^ ^17^ ^]^ with the storage modulus (G’) plateauing gradually at 93 Pa after nearly 3 h (180 min, Figure 1b). The scrFmoc‐GFFRDG peptide had an eight times lower minimum gelation concentration at 0.05% (w/v) and reached the linear viscoelastic plateau (LVE) within 17 min possessing a final G’ of ∼147 Pa. Indicating that even at the significantly lower minimum gelation concentration, 0.05% (w/v) compared to 0.4% (w/v), the scrFmoc‐GFFRDG hydrogel was slightly stiffer than the parent Fmoc‐GFFRGD hydrogel. We postulate here that the rapid self‐assembly of scrFmoc‐GFFRDG results from the negatively charged carboxylic group of aspartic acid sitting adjacent to the positively charged guanidinium of arginine, inducing salt bridge formation.^[^ ^16^ ^]^ As expected from these results, Fmoc‐GFFRGD did not form a hydrogel at 0.05% (w/v), as this is well below its minimum gelation concentration of 0.4% (w/v) under these conditions.

**Table 1:** Summary of the rheological data for the peptide hydrogels studied here. All these studies were carried out in water:DMEM (1:1, w/v, DMEM = Dulbecco's Modified Eagle Medium) at 37 °C.

Parameters	Fmoc‐GFFRGD		scrFmoc‐GFFRDG		Hybrid
^[^ ^a^ ^]^Concentration	0.05%	0.40%	0.05%	0.40%	0.20%/0.20%
^[^ ^b^ ^]^G’[Pa]	No gel	93	147	414	3110
Time at LVE [min]	No gel	435	55	115	201
Strain [%]	No gel	21	3.6	8.7	2.5

^[a]^ As w/v,

^[b]^ G’ = storage modulus.

To directly compare the properties of the two peptide gelators, a rheological time‐sweep measurement was also carried out on scrFmoc‐GFFRDG at 0.4% (w/v) concentration. The storage modulus (G’) was expected to increase significantly with the eightfold increase in concentration compared to the previous experiments at the minimum gelation concentration (0.05%, w/v). However, the gelation process was significantly slower (115 min to reach the LVE), and the storage modulus was only 414 Pa (Figure 1d).

These data suggest that the mechanical properties of the scrFmoc‐GFFRDG hydrogel are not entirely concentration‐dependent.^[^ ^18^ ^]^ However, the resistance to strain increased significantly compared to the 0.05% (w/v) gel sample. The strain‐bearing capacity increased by 2.5‐fold, and the hydrogel broke at 8.7% strain, likely due to a denser network of peptide fibers.

To further explore these peptide gelators, the Fmoc‐GFFRGD and scrFmoc‐GFFRDG were mixed in a ratio of 1:1. This allowed us to probe the contribution of both gelators in a hybrid co‐assembly system, which was designed to have a combined final concentration of 0.4% (w/v). Combining the two peptides resulted in a nearly order‐of‐magnitude stiffer hydrogel, with a final G’ of 3.1 kPa and longer gelation time (∼3.3 h), as well as decreased resistance to stress compared to the individual hydrogels, which had G’ between 91 and 414 Pa at 0.4% (w/v) in their pure form (Figure 1, Table 1).

Based on the above results, we focused our subsequent studies on the two pure gelators at their minimum gelation concentrations: Fmoc‐GFFRGD at 0.40% (w/v), scrFmoc‐GFFRDG at 0.05% (w/v), and the hybrid mixture at 0.20%/0.20% (w/w/v) or a total of 0.4% (w/v) of the two peptide gelators.

**Figure 2:**
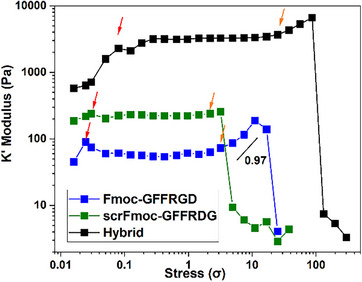
The stiffening response of the three hydrogels to stress is plotted as the differential modulus K′≡δσ/δγ as a function of stress σ at 37 °C in water:DMEM (1:1, v/v, DMEM = Dulbecco's Modified Eagle Medium). Hydrogel samples were measured after being left on a rheometer for long enough to reach a plateau at their linear viscoelastic regime (LVR) at constant f = 1 Hz and γ = 0.2%. The hydrogels were formed at concentrations of 0.40% (w/v), 0.05% (w/v), and 0.20%/0.20% (w/w/v) for Fmoc‐GFFRGD, scrFmoc‐GFFRDG, and the hybrid mixture, respectively.

We next tested the stiffening response of three hydrogels via rheology (Figure 2). Natural biopolymer gels such as collagen^[^ ^19^ ^]^ and fibrin^[^ ^20^ ^]^ exhibit intrinsic stiffening upon stress. This behavior is used by cells to control proliferation, migration, and differentiation within the cellular matrix.^[^ ^21^ ^]^ In some cases, synthetic hydrogels can similarly stiffen when exposed to strain, such as poly(isocyanopeptides), where stem cell differentiation can be controlled by applying strain to the environment.^[^ ^22^ ^]^ The vast majority of known hydrogels capable of strain stiffening (natural or synthetic) are high molecular weight polymers (>80 kDa).

The hexapeptide Fmoc‐GFFRGD mimics the behavior of natural polymers by stiffening under higher stress. The stiffening process occurs in three stages,^[^ ^23^ ^]^ as indicated by the arrows in Figure 2. First, the fiber network partially stiffens within the hydrogel as stress increases, reaching the point marked by the first red arrows. The fibers maintain their structure from the red to orange arrows and align in one direction as strain continues to rise. Beyond the orange arrow, the fibers stretch and resist rupture until the network eventually breaks when they reach their maximum stress resistance. The observation that Fmoc‐GFFRGD does show strain stiffening is noteworthy, given the biological importance of strain stiffening.^[^ ^21^ ^]^ combined with the importance of the RGD ligand in cell adhesion.^[^ ^11^ ^]^ The lack of strain stiffening in scrFmoc‐GFFRDG is equally intriguing, although we acknowledge that this could be a concentration‐dependent factor, and the differences between the two would warrant a separate investigation in future work.

**Figure 3:**
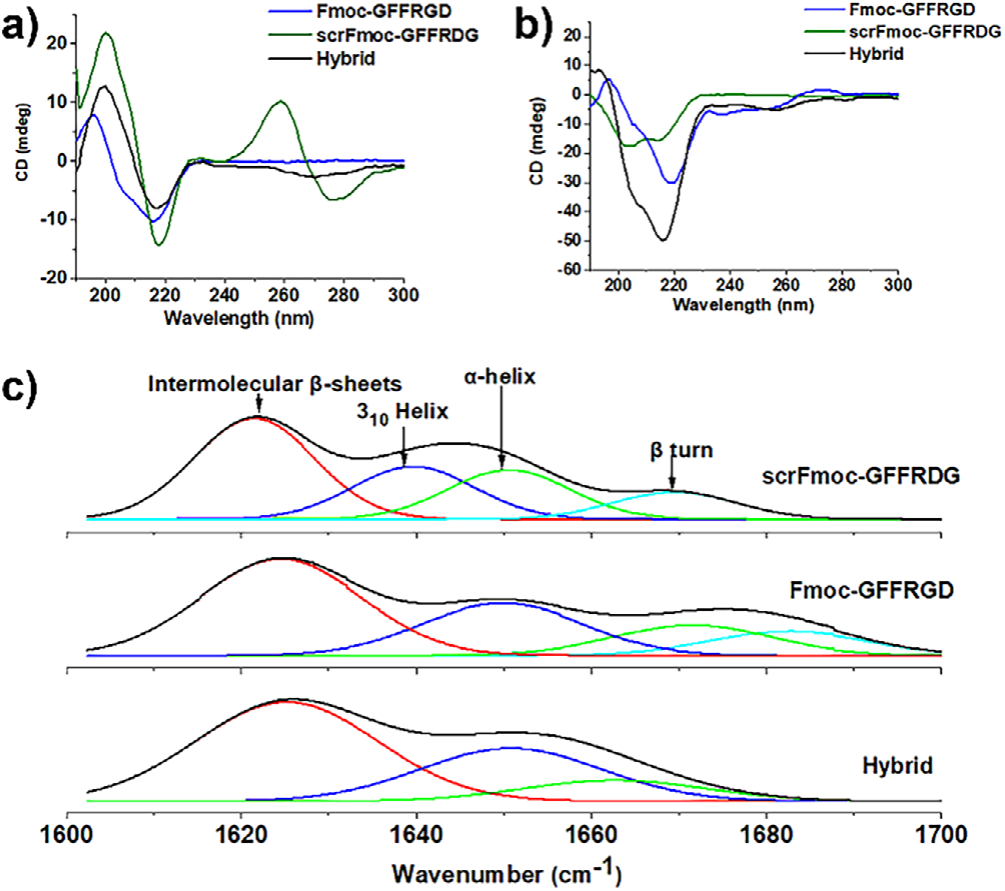
Hexameric peptide secondary structure determination via Circular dichroism (CD) and FT‐IR a) CD Spectra at 25 °C in phosphate saline buffer (PBS) of Fmoc‐GFFRGD (blue line, 4.3 mM), scrFmoc‐GFFRDG (green line, 0.5 mM), and hybrid (black line, 2.15 mM of each peptide; Fmoc‐GFFRGD and scrFmoc‐GFFRDG in total for both peptides is 4.3 mM). b) CD spectra at 37 °C in water:DMEM (1:1, v/v, DMEM = Dulbecco's Modified Eagle Medium) of hydrogels formed from the same hydrogels as in a) and with the same color scheme. In both cases, during the measurement, all samples were diluted two to threefold to avoid the high‐tension (HT) voltage signal saturation. c) Deconvoluted peaks of Fourier transform infrared spectra of hydrogels formed at 25 °C in deuterated water:deuterated PBS (1:1, v/v) mixture showing the selected Amide I region (1600–1700 cm^−1^). The black peak in all cases represents the measured spectra.

Circular dichroism (CD) measurements in a phosphate saline buffer (PBS) at 25 °C revealed further structural differences between the peptide gelators (Figure 3a and Figure S15). The CD spectra for Fmoc‐GFFRGD showed spectral features consistent with β‐sheet structure with minima at 218 nm and maxima at 197 nm^[^ ^17^ ^]^ while the scrFmoc‐GFFRDG CD spectra were reminiscent of helical structures with blue‐shifted minima at 204 and 214 nm, indicating lower helicity (Figure 3a).^[^ ^24^ ^]^ The characteristic peaks of an α‐helix in CD are double minima near 208 and 222 nm.^[^ ^25^ ^]^ The hybrid solution displayed intermediate characteristics, with minima at 215 and 206 nm and a maximum at 193 nm.

The CD measurements were repeated using the hydrogel samples in water:DMEM (1:1, v/v) at 37 °C, followed by two to threefold dilution in the same solvent system (to avoid signal saturation) to investigate the secondary structure of the hydrogels (Figure 3b and Figure S15). The CD spectra of the Fmoc‐GFFRGD and hybrid gels showed similar ellipticity as observed in the solution. However, the CD spectra for the pure scrFmoc‐GFFRDG was shifted significantly, and the new peaks at 260 and 280 nm indicated an altered structure. The two signals at 260 and 280 were ascribed to π → π* transitions in the fluorenyl groups, which suggests that molecular packing of scrFmoc‐GFFRDG in the gel state induces a strong signal in this region due to strong Fmoc‐Fmoc interactions.^[^ ^11b^ ^]^ Furthermore, in the CD spectra of the scrFmoc‐GFFRDG gel, two peaks at 200 and 218 were associated with β‐sheets similar to those seen in Fmoc‐GFFRGD and the hybrid.

Fourier Transform Infrared Spectroscopy (FTIR) was used to investigate the structural properties of the hydrogels further (Figure 3c). The 1600–1700 cm^−1^ region was deconvoluted to reveal the subcomponents within the amide I band. All three hydrogels contain strong peaks at 1620–1624 cm^−1^, characteristic of intermolecular β‐sheets.^[^ ^26^ ^]^ The signals at 1650 cm^−1^ and 1665–1671 cm^−1^ are attributed to α‐helixes and β‐turns. Interestingly, only scrFmoc‐GFFRDG exhibited a peak at 1639 cm^−1,^ which is attributed to 3_10_ helices,^[^ ^27^ ^]^ a structure that generally only occurs in globular proteins containing five or more residues. The helical structure observed in the FT‐IR matches that seen in the solution state CD.

**Figure 4:**
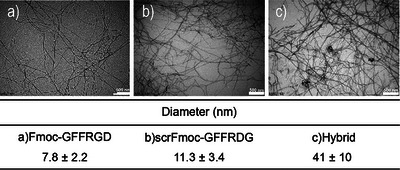
TEM images of hydrogel samples at the indicated concentrations (w/v) that were prepared in water:DMEM (1:1, v/v, DMEM = Dulbecco's Modified Eagle Medium) at 37 °C) and then deposited on a carbon‐coated films and stained with 2% aqueous uranyl acetate and dried before imaging. a) Fmoc‐GFFRGD (0.1%) shows single fiber diameter of 7.8 ± 2.2 nm with minimum fiber diameter of 5.5 nm and maximum 9.9 nm. b) scrFmoc‐GFFRDG (0.1%) with fiber diameter measured as 11.3 ± 3.4 nm with minimum 8 nm and maximum 15 nm diameter. c) Hybrid Fmoc‐GFFRGD/scrFmoc‐GFFRDG (0.1%/0:1%, w/w/v)) with fiber diameter measured at 41 ±10 nm, minimum fiber diameter 32 nm, and maximum 53 nm. Scale bar = 500 nm.

We then used microscopy to determine the morphology of the fibers. The thickness of the fibers was then investigated using Transmission Electron Microscopy (TEM), wherein the samples were stained with uranyl acetate and then dried (see supplementary information for full details). The combination of Fmoc‐GFFRGD and scrFmoc‐GFFRDG peptides in a 1:1 ratio produced gels with fiber thickness of 41 nm, compared to 7.8 and 11.3 nm, for Fmoc‐GFFRGD and scrFmoc‐GFFRDG respectively (Figure 4). The significantly thicker fibers observed for the hybrid gel may explain the nearly tenfold increase in elastic modulus in the resulting hybrid hydrogels compared to the two pure constituent gels at comparable concentrations, as discussed above and shown in Figure 1 and Table 1.

**Figure 5:**
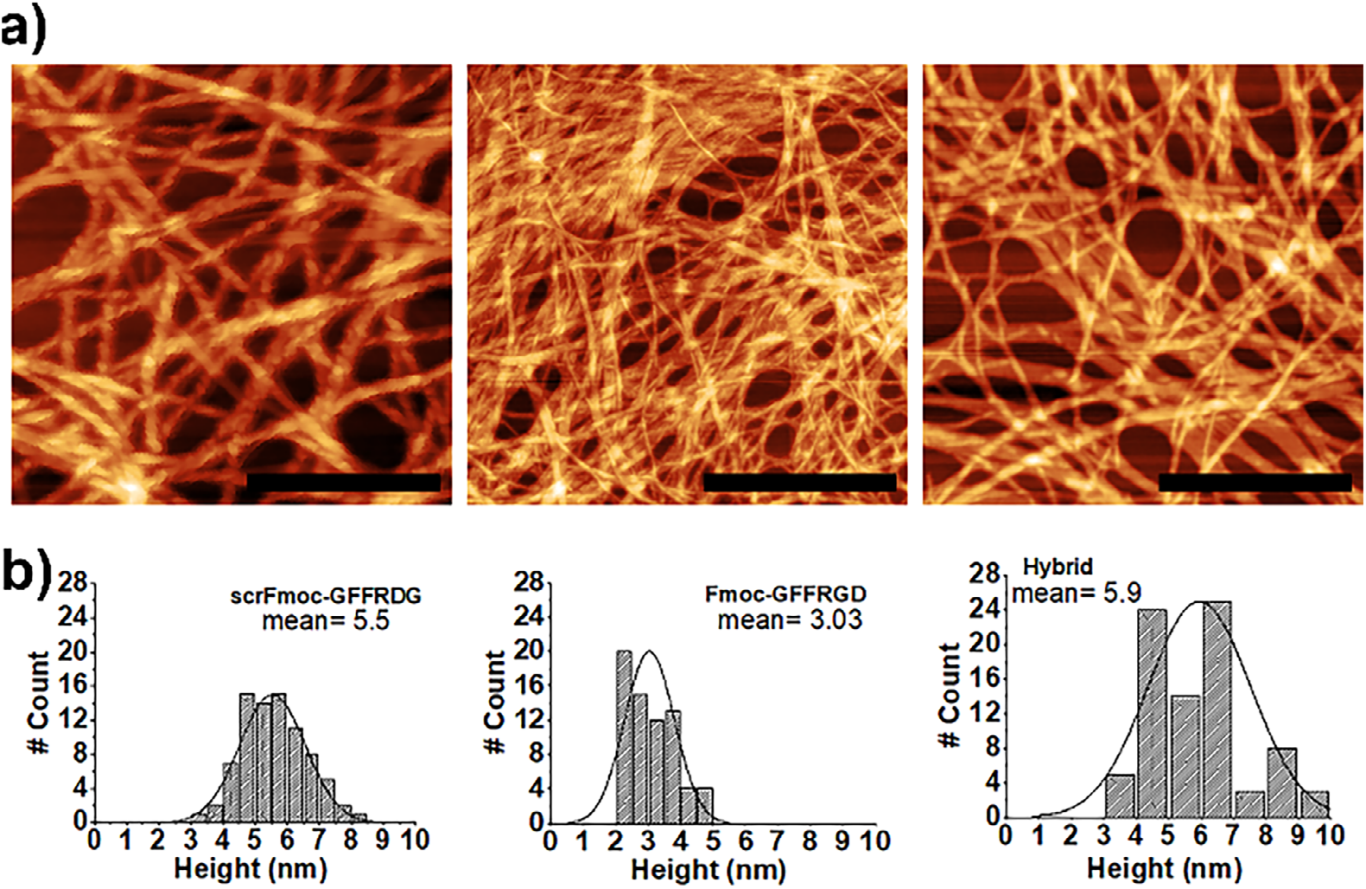
AFM morphology of three peptide hydrogel prepared via pH switch in glucono‐δ‐lactone (GdL)^[^ ^28^ ^]^ Hydrogel solutions were prepared at 0.025% (w/v), at 25 °C casted on freshly cleaved mica, and left overnight to dry. a) Microscopy images of the three hydrogels, scrFmoc‐GFFRDG, Fmoc‐GFFRGD, and Hybrid, in order from left to right. Scale bar = 100 nm, b) The bottom raw height distribution taken from at least 70 measurements with mean calculated 5.5 nm for scrFmoc‐GFFRDG, 3.0 nm for Fmoc‐GFFRGD and 5.9 nm for Fmoc‐GFFRGD/scrFmoc‐GFFRDG (0.025%/0:025%, w/w/v).

While conventional TEM provides only a 2D representation of the fibers, AFM (atomic force microscopy) can provide deeper insight into the morphology through the fiber height. The AFM results correlated well with the TEM results; the Fmoc‐GFFRGD/scrFmoc‐GFFRDG had the thickest fiber of 5.9 nm height, while Fmoc‐GFFRGD and scrFmoc‐GFFRDG fibers were measured to be 3.0 and 5.5 nm high, respectively (Figure 5). In AFM, samples were prepared at a lower concentration (0.025%, w/v) than TEM (0.1%, w/v) and without staining, albeit in a different solvent system (pH switch with glucono‐δ‐lactone; GdL^[^ ^28^ ^]^) to avoid contamination from biological media and inorganic salts found in DMEM and PBS. AFM offers precise height (z‐axis) measurements, but it is less accurate in lateral (x‐ and y‐axis) dimensions due to tip convolution. Both AFM and TEM involve drying the samples before imaging, but AFM samples are prepared at a lower concentration (0.025% w/v) without staining, which likely explain why the absolute magnitude of the diameters measured by AFM and TEM appear different.

### 2.2 2D Cell Viability Assays

**Figure 6:**
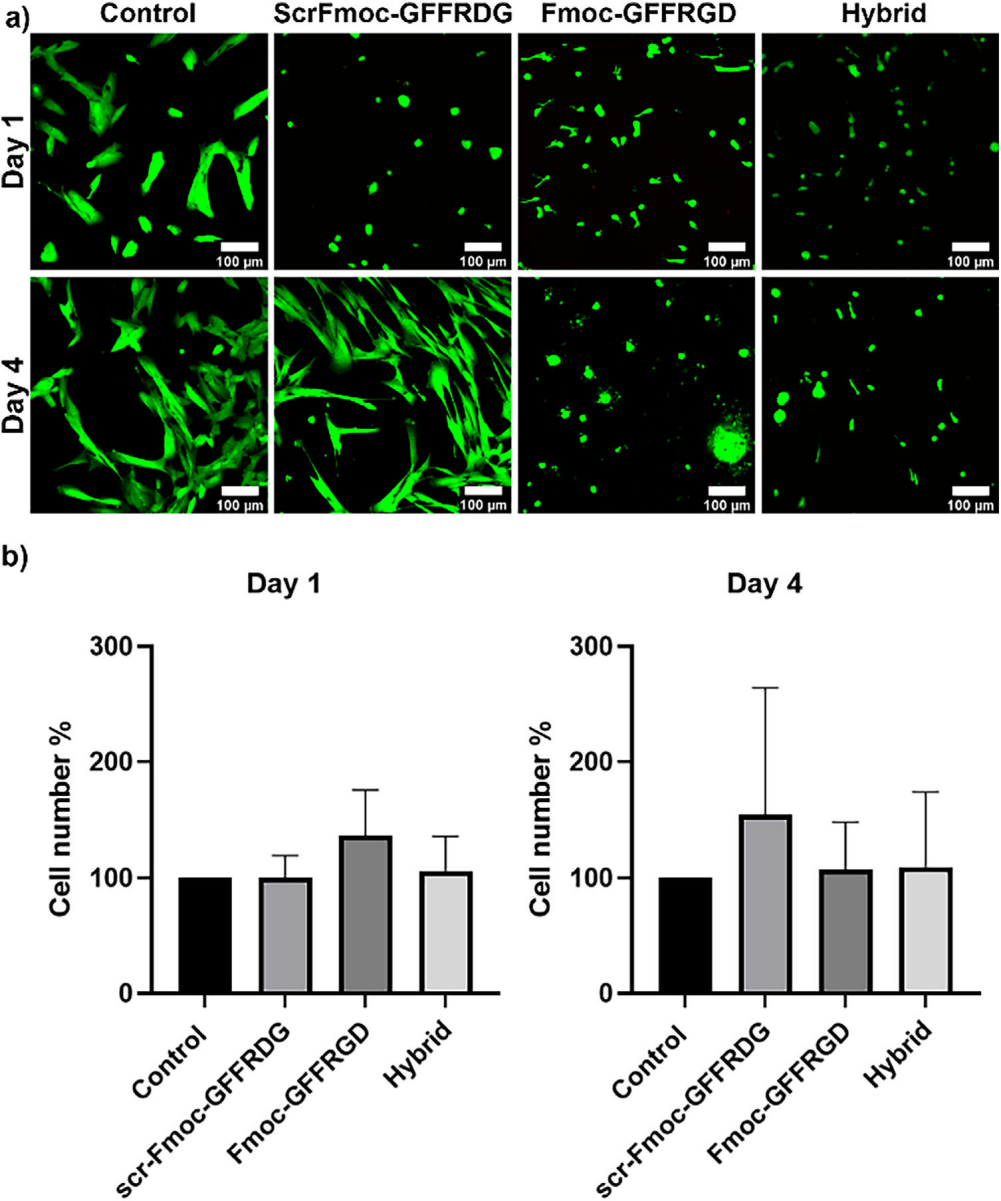
MRC‐5 Fibroblast cultured on top of hydrogels at two time points: day 1 and day 4. a) Live/dead assay images were taken using Zeiss 800 microscope and a control medium. All the gels were formed in 1:1 water:MEME (1:1, v/v, MEME = Minimum Essential Medium Eagle) with the following concentrations of the gelators used: scrFmoc‐GFFRDG (0.05%),Fmoc‐GFFRGD (0.4%), and a hybrid mixture of scrFmoc‐GFFRGD/Fmoc‐GFFRGD (0.2%/0.2%, w/w/v), b) Quantification of cell viability on day 1 and day 4. Error bars denote the standard deviation of the mean (*n *= 4).

Given the role of RGD in cell binding, the viability of the scrambled peptide hydrogen was investigated. We have previously indicated the requirement of RGD for cell binding wherein tripeptide hydrogen – Fmoc‐GFF lacked biocompatibility towards neuroblastoma cells, likely due to the absence of a cell binding motif.^[^ ^1^ ^]^ While RDG is not inherently cytotoxic,^[^ ^29^ ^]^ we wanted to investigate the cell response towards scrFmoc‐GFFRDG. The cytotoxicity of the three gelators was examined by live/dead assays wherein MRC‐5 fibroblasts were cultured on top of hydrogels for 4 days. Calcein AM was used to stain live cells (green) and ethidium homodimer‐1 for dead cells (red, Figure 6). All three gelators were biocompatible and showed no significant toxicity, indicating the cell adhesion motif was not required for viability. A circularity analysis was also completed using the microscopy data, which indicated an elongated cell confirmation (see supporting information, Figure S16).

To investigate the chemical influence of hydrogel composition on cell behavior, we employed antibody staining and confocal imaging to visualize protein expression in embedded cells. This method fixes cells with paraformaldehyde, permeabilizes them with Triton X‐100, and labels α‐smooth muscle actin (α‐SMA) and collagen I with fluorescent antibodies, enabling chemists to correlate biomolecular markers with hydrogel properties.

**Figure 7:**
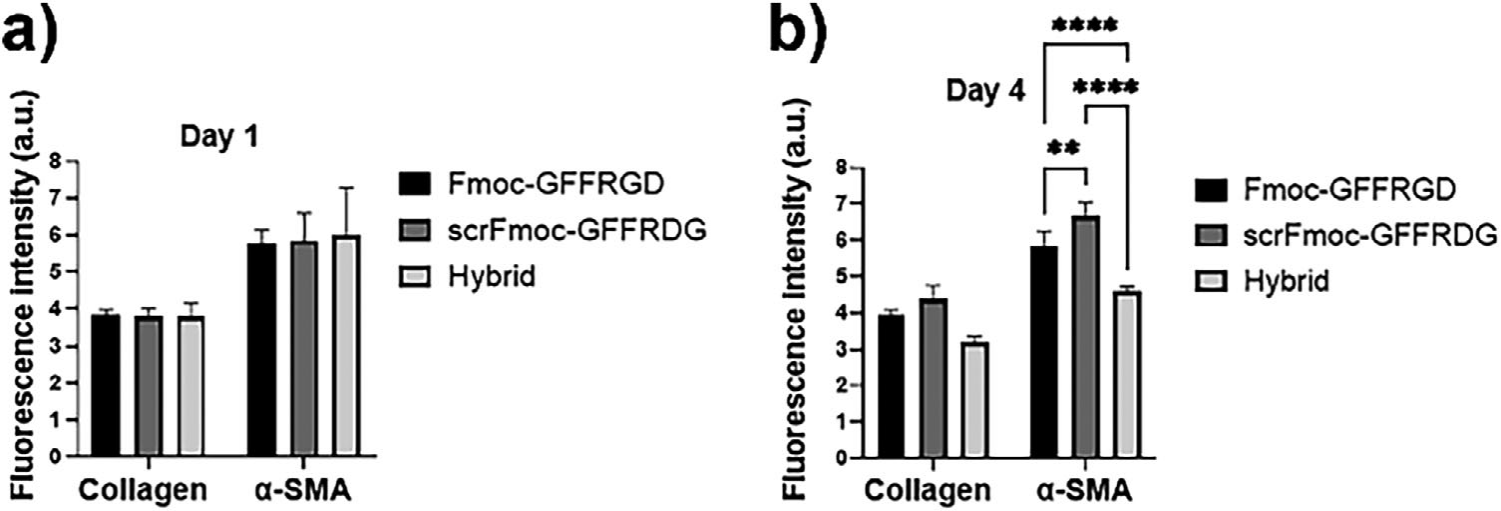
Immunofluorescence studies of pancreatic cancer‐associated fibroblasts (CAF) cultured on top of three peptide hydrogels formed in 1:1 water:IMDM (1:1, v/v, IMDM = Iscove's Modified Dulbecco's Medium) after a) 1 and b) 4 days. Hoechst was used to stain nuclei, Collagen I, and α‐smooth muscle actin (α‐SMA) for ECM production and myofibroblast activation, respectively. Cells were seeded at 100000/mL density.

Given the demonstrated lack of cytotoxicity, further cell culturing was completed to investigate the suitability of the gels for studies of cell behavior. Cancer‐associated fibroblasts (CAF) undergo multiple mechanisms that affect their normal activity, converting them to active CAF. Considerable research has studied the mechanism of CAFs and how inhibition of their activity can restore the immune response against tumors in preclinical trials.^[^ ^29^, ^30^ ^]^ Considering the different morphological characteristics of the MRC5 cells, we hypothesized that scrFmoc‐GFFRDG might influence a CAF phenotype, monitored by α‐smooth muscle actin (α‐SMA) and collagen I expression.^[^ ^31^ ^]^ To test this, we cultured CAFs on top of the hydrogels and tracked the expression of α‐SMA and collagen 1. (Figure 7). On day one, α‐SMA expression was slightly higher in the hybrid gel than in the single peptide gels, though not significantly. By day four, both proteins were reduced in the hybrid gel but increased in the scrFmoc‐GFFRDG gel. These results suggest a correlation between hydrogel stiffness and the expression of markers associated with a CAF phenotype.

While the presence of RGD in a 3D matrix is well established to support cellular processes like adhesion, migration and proliferation, quantification of specific integrin‐RGD motifs in our system has proved challenging due to the soft matrix used. Furthermore, because the scrambled peptide promotes a very different hydrogel microenvironment than that fostered with the RGD, there is not a suitable negative control to quantify adhesion between the two systems. Therefore, the varying phenotypes between the two systems are likely due to multiple factors, including porosity, stiffness, and nanoarchitecture.

## 3 Conclusion

In conclusion, we developed the peptide scrFmoc‐GFFRDG by scrambling the previously reported Fmoc‐GFFRGD,^[^ ^17^ ^]^ resulting in significantly faster gelation kinetics at lower minimal gelation concentrations (0.05% vs 0.4% w/v). Rheological, AFM, and TEM analyses revealed that scrFmoc‐GFFRDG forms stiffer hydrogels with thicker fiber structures. Interestingly, these differences seem to be related to the slight change in the relative placement of positive and negative charges on the RGD versus RDG C‐terminus of these two peptides. FT‐IR and CD confirmed the changes in the secondary structure present in the scrambled peptide hydrogel compared to the parent sequence. These are attributed to the altered peptide interactions, most likely due to the subtle differences in electrostatic interactions. How subtle changes in charge distribution on the C‐terminus of the peptide, on going from RGD to the RDG motif of this peptide, influence the differences in Fmoc‐group interactions evident in the CD spectra, would be an interesting topic for future investigation.

Cytotoxicity assays confirmed that fibroblast cells remained viable when cultured in scrFmoc‐GFFRDG, and culturing CAF on these hydrogels showed a slight increase in α‐SMA expression, indicating a gel‐promoting preferential CAF activity. This study demonstrates the ability of sequence‐engineered peptide hydrogels to develop finely tuned 3D cell cultures and shows that traditional binding motifs are not essential for cell compatibility–on the contrary, a slight modification to a naturally occurring binding motif may result in superior biomaterials for cell culture applications.

## 4 Experimental

### Peptide synthesis and purification

Peptide synthesis was conducted using Fmoc solid‐phase peptide synthesis, either manually or on an automated synthesizer. The amino acids were used as received from the vendor. Purification was performed using high‐performance liquid chromatography (HPLC) with a reversed‐phase C18 column (22 mm diameter, 150 mm length). Further details on the characterization are provided in the supporting information.

### Hydrogel assembly

The peptide hydrogels were formed by dissolving the peptide in MilliQ water, then adding sodium hydroxide (0.1 M), and lastly adding Dulbecco's Modified Eagle Medium (DMEM, Gibco) at a 1:1 volume ratio. The 0.4% (w/v) hydrogels were prepared by dissolving the peptide in Milli‐Q water with 0.1 M sodium hydroxide (1 equivalent) and then mixing it with an equal volume of DMEM cell culture media. The final concentration of the gelator was 4.3 mM. For 0.05% (w/v) scrFmoc‐GFFRDG, Milli‐Q water and 0.1 M sodium hydroxide (1.2 equivalents) were added, followed by DMEM in a 1:1 volume ratio, resulting in a final concentration of 0.5 mM. Both gelators were left at room temperature overnight to form the gels.

### Circular dichroism

Characterization was conducted using an Applied Photophysics Chirascan Plus CD spectrometer with parameters set to a wavelength range of 190–300 nm, step size of 1 nm, bandwidth of 1 nm, and a 1 mm pathlength quartz cuvette. The instrument was purged with nitrogen for 1 hour, and the lamp was pre‐warmed for 30 minutes prior to recording measurements. Samples of the peptides were prepared at the following concentrations for Fmoc‐GFFRGD (0.4%, w/v, 4.3 mM), r scrFmoc‐GFFRDG (0.05%, w/v, 0.5 mM) and hybrid Fmoc‐GFFRGD/scrFmoc‐GFFRDG (0.2%/0.2%, w/w/v, 2.15 mM of each) in either a) phosphate saline buffer (PBS at pH = 7.0) or in 1:1 water:DMEM using the same sample preparation methods as above for hydrogel assembly. Samples were left to settle overnight, and in both cases, diluted two to threefold during measurements to keep high voltage (HV) below 1000.

### FT‐IR

Peptide gels were prepared at 10 mg mL^−1^ in deuterated water (D_2_O). Gelators (20 mg mL^−1^) were dissolved in NaOD (0.1 M NaOD in D_2_O), followed by an equal volume addition of phosphate saline buffer (PBS) that had been freeze‐dried and reconstituted in D_2_O, resulting in a 1:1 D_2_O:PBS (v/v) mixture. Samples were characterized using a Cary 630 with parameters set to 16 scans, 4 cm^−1^ resolution, and a scan range of 400–4000 cm^−1^. Deconvolution was completed using OriginLab software to fit the curves assuming Gaussian shaped bands.

### Atomic force microscopy (AFM)

AFM analysis was performed on a Bruker Multimode 8 in ScanAsyst mode. The morphology of the peptide gelators was analyzed using tapping mode AFM. The peptide gelators were examined in their gel state, and scrFmoc‐GFFRDG was also analyzed in solution. The individual peptide gels (Fmoc‐GFFRGD, 0.025%, w/v and scrFmoc‐GFFRDG, 0.025%, w/v) were prepared by dissolving gelators in Milli‐Q water and 0.1 M NaOH, followed by addition of glucono‐δ‐lactone (GdL).^[^ ^28^ ^]^ Hybrid gelator Fmoc‐GFFRGD/scrFmoc‐GFFRDG (0.025%/0.025%, w/w/v) was prepared similarly. Solutions were diluted to 0.1 mg mL^−1^ (individual gelators) or 0.2 mg mL^−1^ (hybrid) before casting 10 µL onto freshly cleaved mica substrates. Samples were air‐dried overnight at room temperature

### Cell culture

MRC‐5 fibroblast cells were cultured in Minimum Essential Medium Eagle (MEME, Sigma Aldrich), supplemented with 10% fetal bovine serum and 1% penicillin/streptomycin. Media was changed every 2–3 days and cells passaged at 80–90% confluency.

### Cancer‐associated fibroblasts

Primary murine KPC, CAFs, were cultured in Iscove's Modified Dulbecco's Medium (IMDM, Gibco) without phenol red, supplemented with 10% fetal bovine serum and 4 mM GlutaMAX. Cells were lifted with 0.05% trypsin and passaged at 80–90% confluency, with media refreshed every two days.

### 2D live dead assay

Peptides were sterilized under UV light for 10 min before the assay. Hydrogels were prepared as described above in 1:1 water:DMEM, substituting DMEM with MEME. An aliquot of each gelator (50 µL) was deposited in triplicate into 96‐well plates. After 24 h, MRC‐5 fibroblasts at 100 000 cells mL^−1^ were added to each gelator sample. Control cells were added to untreated wells.

### LIVE/DEAD stain

Calcine‐AM (live)/Ethidium homodimer‐1(dead) dye (catalog number: L3224) was prepared according to the manufacturer's protocol. Whereby, calcein‐AM (0.5 µL) and ethidium homodimer‐1 (2 µL) were diluted in 1 mL of PBS (tissue‐culture grade). Once formulated, 50 µL of the live/dead solution was added to each well, and the plate was incubated for 40 min at 37 °C. Wells were then washed with Dulbecco‘s phosphate‐buffered saline (DPBS) to remove excess dye, and cells were refreshed with new media every other day.

### Antibody staining and imaging

Embedded cells were fixed with 4% paraformaldehyde (Sigma, USA) for 20 min then permeabilized with 0.1% Triton X‐100 (Fisher, USA) in PBS for 30 min. After washing, the samples were blocked with 1% bovine serum albumin (BSA) in PBS for 15 min. Primary antibodies α‐smooth muscle antibody mouse monoclonal (1:400, Sigma‐Aldrich, USA) and anti‐collagen I, rabbit polyclonal (1:200, ab34710 abcam, USA) were prepared in 1% BSA in PBS and added to the samples for 1 h at room temperature. Secondary antibody goat anti‐mouse IgG (H + L), Alexa Fluor 647 (1:200, ThermoFisher, USA) and donkey anti‐rabbit IgG (H + L) highly cross‐absorbed Alexa Fluor 555 (1:200, ThermoFisher, USA) anti‐phalloidin 488 (1:200, Sigma‐Aldrich, USA), and Hoechst (1:300) were added to 1% BSA in PBS for 1 h. Cell Discoverer 7 LSM 900 (Carl Zeiss, Germany) inverted Zeiss laser scanning confocal microscope 900 with 5X (N.A. 0.35, air immersion, W.D. 5.10 mm) with 2X afocal magnification changer was used to image.

## Supporting Information

The authors have cited additional references within the Supporting Information.^[^ ^32^ ^]^

## Conflict of Interests

The authors declare no conflict of interest.

## Supporting information



Supporting information

## Data Availability

The data that support the findings of this study are available in the supplementary material of this article.;
