# Supplementary material for: Scrambled RGD Hexameric Peptide Hydrogel Supports Efficient Self‐Assembly and Cell Activity
Source: Chemistry. 2025 Apr 21;31(27):e202404410. doi: 10.1002/chem.202404410 (PMC12080297; doi:10.1002/chem.202404410)
Supplement: Supplementary file 1 — Supporting information [file CHEM-31-e202404410-s001.docx]

Electronic Supporting Information

Scrambled RGD hexameric peptide hydrogel supports efficient self-assembly and cell activity

Karrar Al Taief^[a,b]^, Stephanie Nemec^[c,d]^ Isis A. Middleton^[a,b]^ Kristopher A. Kilian,^[c,d]^ Pall Thordarson^[a,b,d]*^

^[a]^ Dr. K. A. Taief, Dr. I. A. Middleton, Prof. Dr. P. Thordarson

School of Chemistry, University of New South Wales

Sydney, NSW, 2052 Australia

E-mail: p.thordarson@unsw.edu.au

^[b]^ Dr. K. A. Taief, Dr. I. A. Middleton, Prof. Dr. P. Thordarson

The UNSW RNA Institute, University of New South Wales

Sydney, NSW, 2052 Australia

^[c]^ Dr. S. Nemec, Prof. Dr. K. Kilian

School of Materials Science and Engineering

University of New South Wales

Sydney, NSW, 2052 Australia

^[d]^ Dr. S. Nemec, Prof. Dr. K. Kilian

The Australian Centre for Nanomedicine, University of New South Wales

Sydney, NSW, 2052 Australia

Table of Contents

S1. General 3

S1.1 Materials 3

S1.2 Instrumentation 3

S1.2.1 Semi-preparative HPLC 3

S1.2.2 Low resolution Electrospray Ionisation mass spectra (ESI-MS) 3

S1.2.3 Reversed-Phase Liquid chromatography–mass spectrometry (LC-MS) 3

S1.2.4 Nuclear Magnetic Resonance (NMR) 3

S1.2.5 Rheology 3

S2. Peptide synthesis 4

S2.1 scrFmoc-GFFRDG 4

S2.1 Fmoc-GFFRGD 8

S3. Supplementary Figures 11

# General

## Materials

All the Fmoc-capped amino acids; Fmoc-glycine, Fmoc-Phe-OH, Fmoc-(2,2,4,6,7-pentamethyldihydro-benzofuran-5-sulfonyl)- l-arginine and Fmoc-l-aspartic acid-tert-butyl ester were used as received without future purification from ChemImpex. 2-Chlorotrityl chloride resin (1.0 - 1.6 mmol/g, 100 - 200 mesh, ChemImpex catalogue number: 03498) was used in solid-phase peptide synthesis (SPPS). DMEM was obtained from Gibco with other reagents and solvents purchased from Merck. SPPS was completed either manually on an orbital shaker or on an automated Biotage Initiator^+^ Alstra.

## Instrumentation

### Semi-preparative HPLC

Purification was carried out on Shimadzu Prominence UFLC HPLC system equipped with a LC-20AB pump and fraction collector. Detection was made using SPD-20A PDA detector at 254 nm. The mobile phase consisted of eluents A (0.1% aqueous formic acid) and B (0.1% formic acid in acetonitrile). Vision HT C18 HL 5 μ 150 x 22 mm semi-preparative column was used as the stationary phase at a flow rate of 5 mL/min. Data acquisition and data analysis was performed on Lab Solution Version 5.8. The mobile phase gradient was set to (B) as eluent: *t* = 0 – 5 minutes 15% B; *t* = 5 – 13 minutes gradient 15-60 % B; *t* = 13– 25 minutes 60% B, *t* = 25-27 minutes 15% gradient B (at 254 nm).

### Electrospray Ionisation mass spectrometry (ESI-MS)

High resolution mass spectrometry was performed using an Thermo LTQ Orbitrap XL instrument. Low resolution ESI-MS were recorded on a Waters Micromass® ZQ™ mass spectrometer with MassLynx processing software or a Thermo Scientific LCQ Fleet Ion Trap mass spectrometer equipped with Xcalibur processing software.

### Reversed-Phase Liquid chromatography–mass spectrometry (LC-MS)

LC-MS spectra were recorded on Shimadzu Prominence Ultra-Fast Liquid Chromatography (UFLC) High Performance Liquid Chromatography (HPLC) system equipped with LC-20AD pump and a 2010 EV LCMS detector. X-BridgeTM C18 column 5 μm, 4.6 x 150 mm was used as a stationary phase with a flow rate of 0.3 mL/min. The mobile phase consisted of eluents A: 0.1% (v/v) aqueous formic acid and B 0.1% (v/v) formic acid in acetonitrile.

### Nuclear Magnetic Resonance (NMR)

Spectroscopy spectra were recorded on 400 MHz Bruker Avance III NMR system fitted with a BBFO. All samples were dissolved in DMSO-*d*_6_ with signals referenced to their respective residual solvent peaks (DMSO-*d*_6_: ^1^H =2.50 ppm and ^13^C = 39.52 ppm). Signals were recorded in the following order where relevant: chemical shift (ppm), identified integral, multiplicity, coupling constants (*J* in Hz) and assignments. All NMR data were processed using MestReNova 14.2 Software. Multiplicities are assigned as singlet (s), doublet (d), triplet (t), quartet (q), pentet (p), sextet (sx), doublet of doublet (dd), doublet of triplet (dt), triplet of doublet (td), multiplet (m), or broad (br).

### Rheology

Anton Paar MCR 302 Rheometer equipped with a 25 mm stainless-steel parallel plate and water pump for temperature control was used for the rheological measurements. The data was analysed with RheoPlus v3.61 software. For typical rheology measurements, 600 μL of freshly prepared gel was casted onto the stainless-steel plate. Time sweep was completed at 37 °C with constant 0.2% strain and 1 Hz frequency. Frequency sweeps were set to a logarithmic ramp starting from 10 Hz to 0.1 Hz with constant 0.2% strain. Data points were collected every 2 seconds over 5 minutes. Measurements were recorded in triplicate and the average was plotted. Strain sweeps were performed with constant frequency (f) = 1 Hz, log ramp strain (γ) = 0.1 – 100% and measurements taken every 2 seconds for 5 minutes.

For stress stiffening the protocol was adapted from literature.^[1]^ A total of 49 measurements were recorded, with relaxation intervals of 180 seconds and stress intervals of 150 seconds. During each relaxation interval the oscillatory shear stress with constant frequency (τ) was set to be 1/10^th^ of the shear stress. At each stress interval, shear stress was averaged and divided by 100. Differential modulus (*K*’) was calculated as average of shear stress divided by shear strain *K*=*d*σ/*d*γ and plotted against stress (σ) (0.016 - 1009 Pa).

# Peptide synthesis

Peptides were synthesised in the C to N terminus direction. Fmoc-GFFRGD was synthesised as described previously.^[2]^ scrFmoc-GFFRDG was synthesised as described in detail below. 400 mg of 2-chlorotrityl chloride resin (loading =1.0 to 1.1 mmol/g) was weighed into a 10 mL fritted polypropylene syringe. Resin was swelled by 5 mL dichloromethane (3 times for 10 minutes) on orbital shaker. Fmoc-Gly-OH (3 eq, 0.392.4 g) was dissolved in *N,N*-dimethylformamide (2 mL), dichloromethane (2 mL), and 8 equivalents of diisopropylethylamine (0.6 mL), and added to the resin and stirred overnight on the shaker.

Before deprotecting, the peptide-resin solution was washed with *N*,*N*-dimethylformamide (3 times for 3 minutes). The deprotection was carried out by adding 20% (v/v) of piperidine in *N*,*N*-dimethylformamide for 5 minutes (5 mL) and then for 10 minutes (5 mL).Subsequent coupling was done by dissolving the amino acids in the coupling reagent mixture made from 1-hydroxybenzotriazole hydrate (HOBT, 1.7 g) and *O*-(Benzotriazol-1-yl)-*N*,*N*,*N*',*N*'-tetramethyluronium hexafluorophosphate (HBTU, 4.7 g) in *N*,*N*-dimethylformamide (0.5 M of each reagent, 25 mL) and 6 equivalents of *N,N*-diisopropylethylamine (0.46 mL) and adding it to peptide-resin solution. The deprotection, washing and coupling repeated for Fmoc-l-aspartic acid-*tert*-butyl ester (3 eq. 0.543 g), Fmoc-Arg (3 eq. 0.856 g), Fmoc-Phe-OH (3 eq. 0.511 g) and Fmoc-Gly-OH (3 eq. 0.392.4 g). After the last coupling of amino acid while leaving the Fmoc group on, the resin was washed with *N*,*N*-dimethylformamide for 3 minutes, dichloromethane for 3 minutes, and methanol (3 times for 1 minute) before cleavage. The peptide was then cleaved off the resin using trifluoracetic acid, triisopropyl silane, and water (95:2.5:2.5, v/v), and the mixture was stirred for 3 hours on a shaker, and the cleavage solution was collected in a round bottom flask. The resin was then washed with dichloromethane (3 times for 5 minutes) and added to the crude solution and then it was concentrated by rotary evaporation. The crude peptide was then dissolved in water/acetonitrile (30:70, v/v) and purified immediately by semi-preparative HPLC.

## scrFmoc-GFFRDG





Purification of crude scrFmoc-GFFRDG was done on semi-preparative HPLC. Purity acquired >99% calculated from chromatogram at 254 nm and the yield was 78.9 mg or 26% as white fluffy powder. ^1^H NMR (400 MHz, DMSO-*d*_6_) δ 9.38 (s, 1H), 8.61 (d, J = 8.5 Hz, 1H), 8.18 (d, J = 8.1 Hz, 1H), 8.08 (d, J = 7.8 Hz, 1H), 7.97 (d, J = 8.3 Hz, 1H), 7.89 (d, J = 7.5 Hz, 2H), 7.70 (d, J = 7.5 Hz, 2H), 7.50 (t, J = 6.1 Hz, 1H), 7.41 (t, J = 7.4 Hz, 2H), 7.32 (t, J = 7.4 Hz, 2H), 7.24 (d, J = 6.3 Hz, 3H), 4.49 (td, J = 8.7, 4.2 Hz, 1H), 4.34 (t, J = 6.9 Hz, 1H), 4.24 (s, 1H), 3.58 (d, J = 6.1 Hz, 1H), 3.06 – 2.90 (m, 2H), 2.85 – 2.77 (m, 1H), 2.69 (td, J = 15.4, 7.8 Hz, 2H), 2.44 (dd, J = 16.4, 7.4 Hz, 1H), 1.56 (s, 3H). ^13^C NMR (151 MHz, DMSO-*d*_6_) δ 172.11, 171.18, 170.57, 168.79, 157.25, 156.42, 143.84, 140.71, 137.64, 129.26, 129.22, 128.94, 128.07, 127.97, 127.65, 127.31, 127.10, 126.22, 125.28, 120.12, 65.79, 46.59. IR (ATR):3276(m), 1626(s), 1539(s), 1392(w), 1252(m). HRMS (ESI^+^) *m/z*: [M+H]^+^ calculated for C_47_H_54_N_9_O_11_ is 920.3942. found: 920.3934.

Figure S1. HPLC trace of purified scrambled peptide scrFmoc-GFFRDG.

Figure S2. Low resolution mass spectrometry (ESI) of scrFmoc-GFFRGD. The main ion is for [M+H]^+^ at 920.5 m/z.


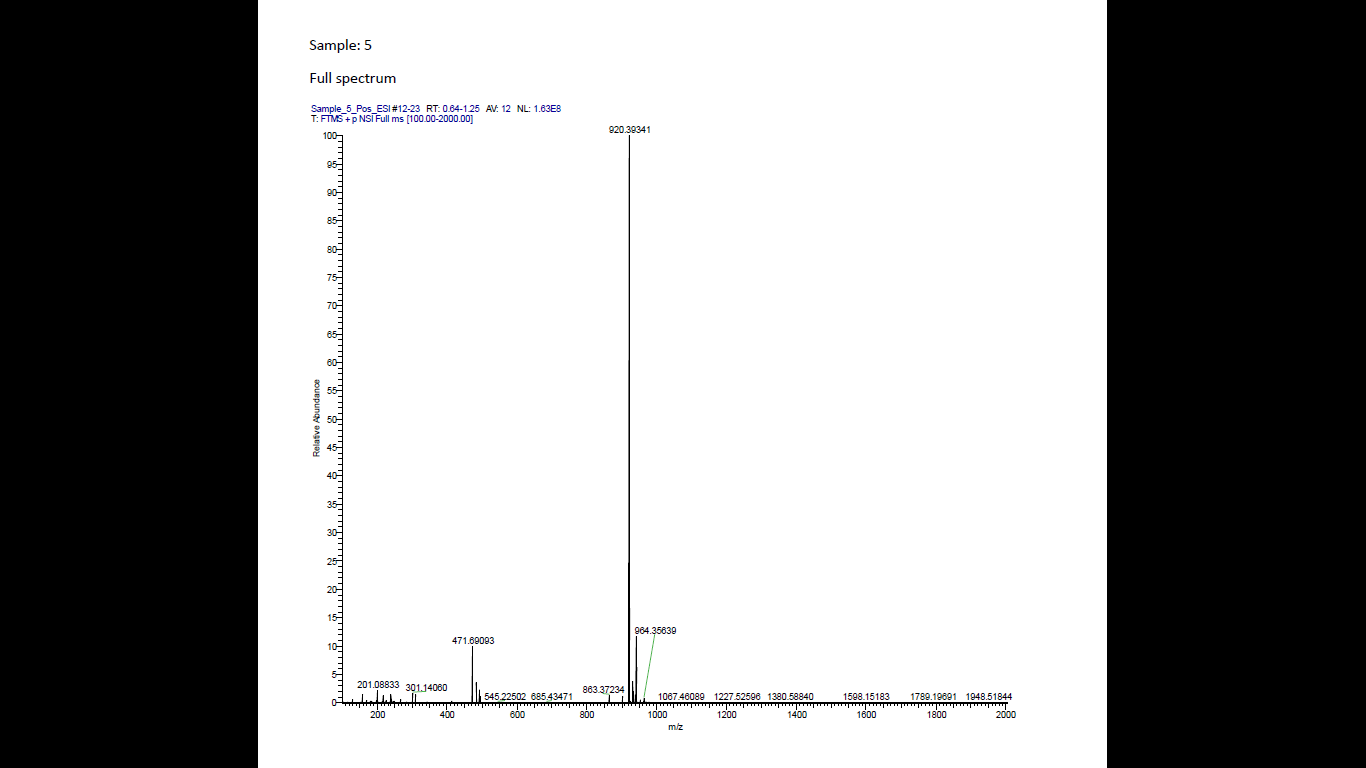


Figure S3. High resolution mass spectrometry (ESI) of scrFmoc-GFFRDG. The main ion is for [M+H]^+^ at 920.3934 *m/z*.


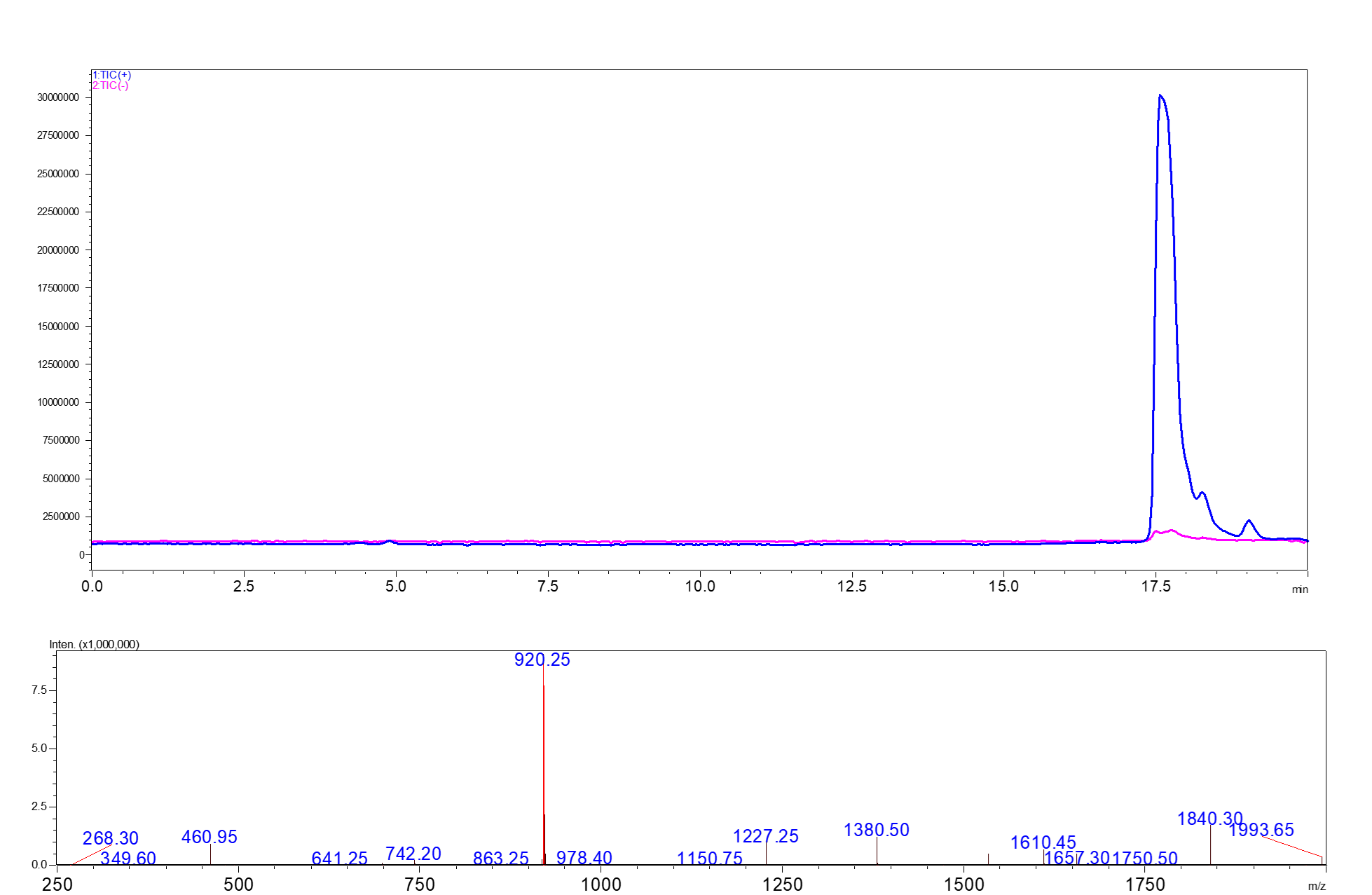


Figure S4. LCMS trace of scrFmoc-GFFRDG. Top) Total ion count and bottom) MS trace for the main peak at *c.a.* 17.5 min. The main ion is for [M+H]^+^ at 920.2 *m/*z.


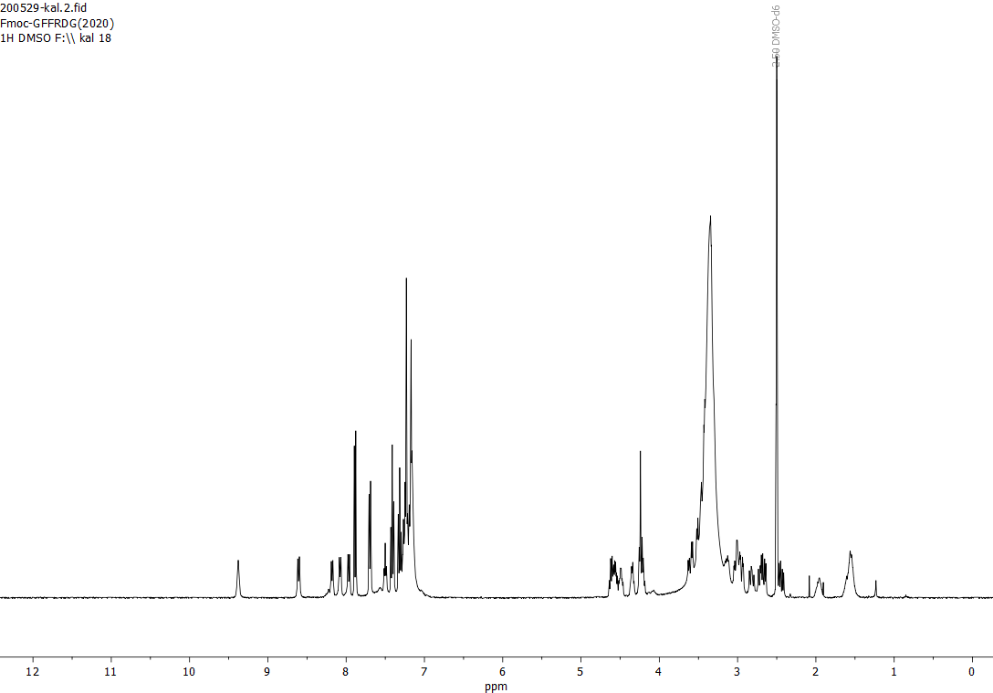


Figure S5. ^1^H NMR spectra (400 MHz, DMSO-*d*_6_) of scrFmoc-GFFRDG.


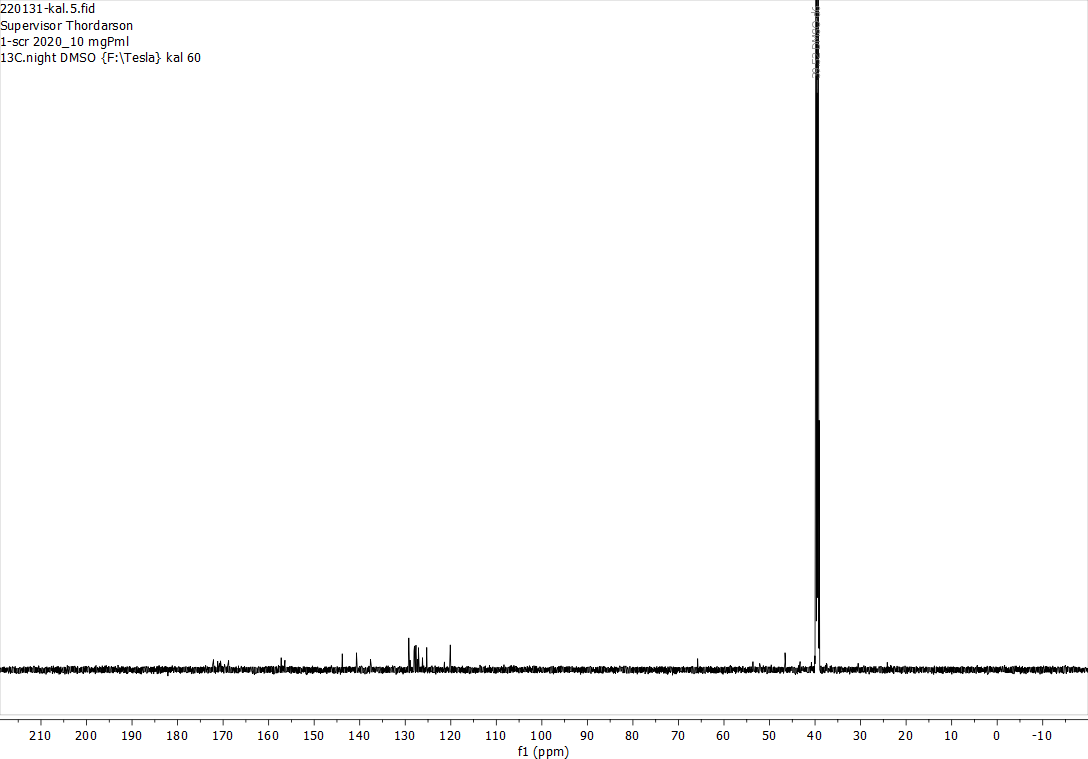


Figure S6. ^13^ C NMR spectra (151 MHz, DMSO-*d*_6_) of scrFmoc-GFFRDG.


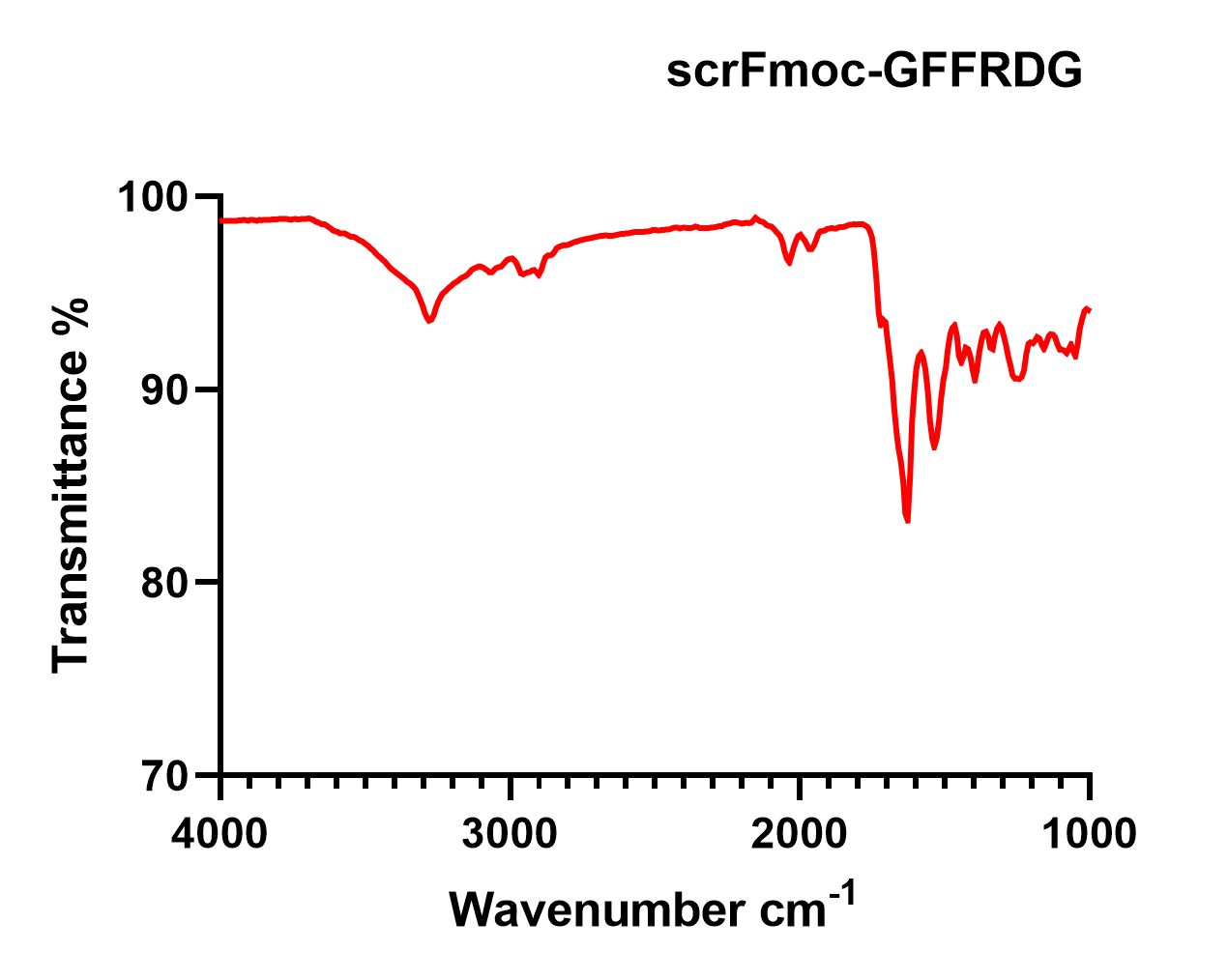


Figure S7. Spectra of scrFmoc-GFFRDG (solid).

## Fmoc-GFFRGD

**

**

The crude Fmoc-GFFRGD was synthesized according to our previously published method^[2]^ and purified using semi-preparative HPLC. Purity acquired >99% calculated from chromatogram at 254 nm and the yield of Fmoc-GFFRGD was 100 mg, 25%.

^1^H NMR (400 MHz, DMSO-*d*_6_) δ 9.14 (s, 1H), 8.48 (d, J = 6.3 Hz, 1H), 8.26 (d, J = 7.8 Hz, 1H), 8.22 – 8.15 (m, 2H), 8.12 (d, J = 7.5 Hz, 1H), 8.00 – 7. 91 (m, 2H), 7.88 (d, J = 7.6 Hz, 5H), 7.84 (d, J = 6.8 Hz, 1H), 7.69 (d, J = 7.5 Hz, 4H), 7.58 (d, J = 5.9 Hz, 1H), 7.48 (q, J = 5.6 Hz, 2H), 7.41 (t, J = 7.4 Hz, 4H), 7.31 (t, J = 7.4 Hz, 4H), 7.25 (dd, J = 5.7, 4.1 Hz, 8H), 7.17 (dt, J = 10.9, 7.5 Hz, 13H), 4.63 – 4.41 (m, 5H), 4.35 – 4.09 (m, 10H), 3.94 (dd, J = 16.9, 7.3 Hz, 1H), 3.62 (d, J = 6.2 Hz, 1H), 3.58 (d, J = 6.1 Hz, 1H), 3.50 (t, J = 5.6 Hz, 1H), 3.46 (t, J = 5.6 Hz, 1H), 2.88 – 2.77 (m, 2H), 2.70 (dd, J = 13.9, 9.3 Hz, 2H), 1.74 (d, J = 8.4 Hz, 1H), 1.52 (s, 4H). ^13^C NMR (101 MHz, DMSO-*d*_6_) δ 171.68, 170.79, 168.02, 166.36, 160.31, 157.11, 143.82, 140.71, 137.64, 129.21, 128.06, 127.97, 127.64, 127.09, 126.21, 125.26, 120.11, 65.78, 53.71, 46.59, 40.71, 37.55, 30.12, 26.12. IR (ATR): 3274 (m),3063 (w),1630(s),1539(s),1390(w), 1255(m). Low resolution (ESI+) *m/z*: [M+H]^+^ calculated for C_47_H_54_N_9_O_11_ is 920.39 found: 920.58 This data is in good agreement with previous reports.^[2]^

Figure S8. HPLC trace of purified Fmoc-GFFRGD peptide


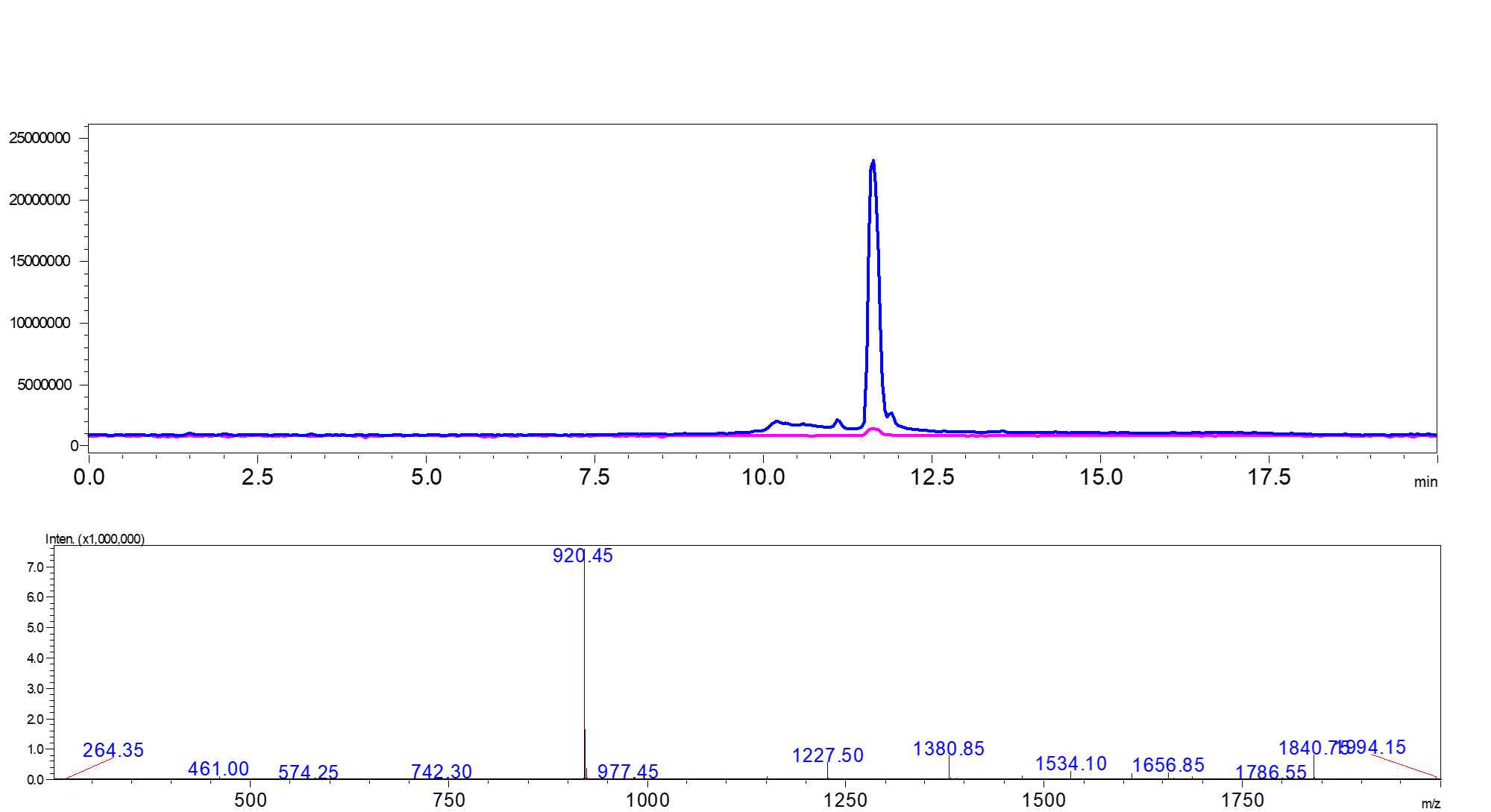


Figure S9. LCMS trace of Fmoc-GFFRGD. Top) Total ion count and bottom) MS trace for the main peak at *c.a.* 17.5 min. The main ion is for [M+H]^+^ at 920.2 *m/*z.

Figure S10. Low resolution mass spectrometry (ESI) of Fmoc-GFFRGD. The main ion is for [M+H]^+^ at 920.5 *m/z*.


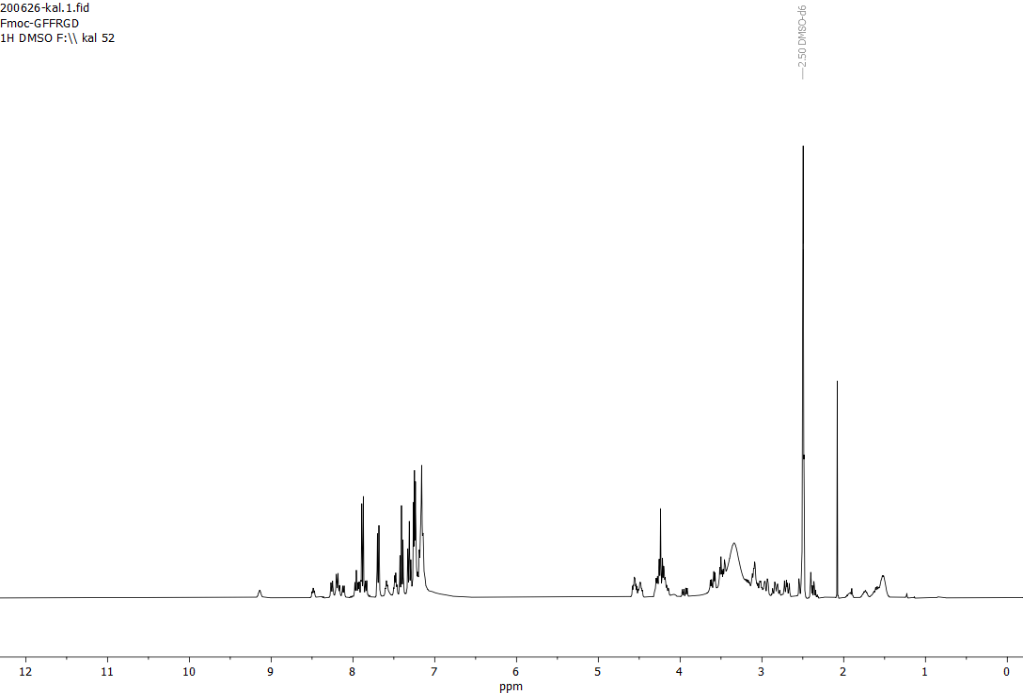


Figure S11. ^1^H NMR spectra (400 MHz, DMSO-*d*_6_) of Fmoc-GFFRGD.


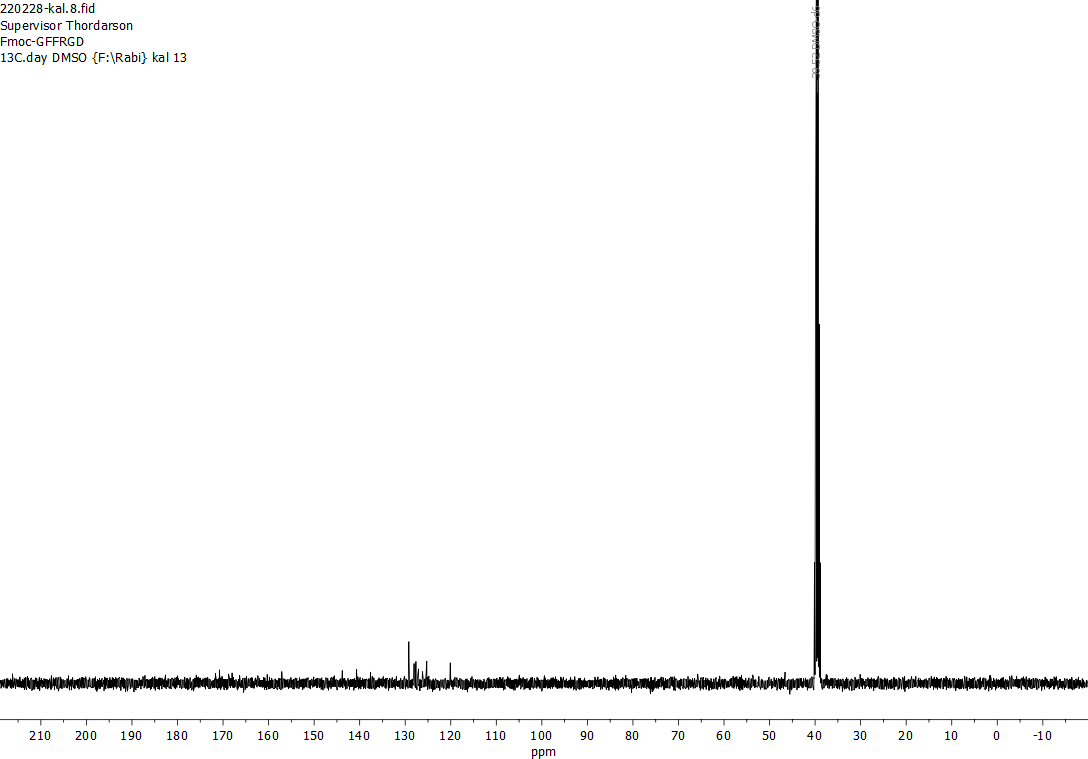


Figure S12. ^13^C NMR spectra (101 MHz, DMSO-*d*_6_) of Fmoc-GFFRGD


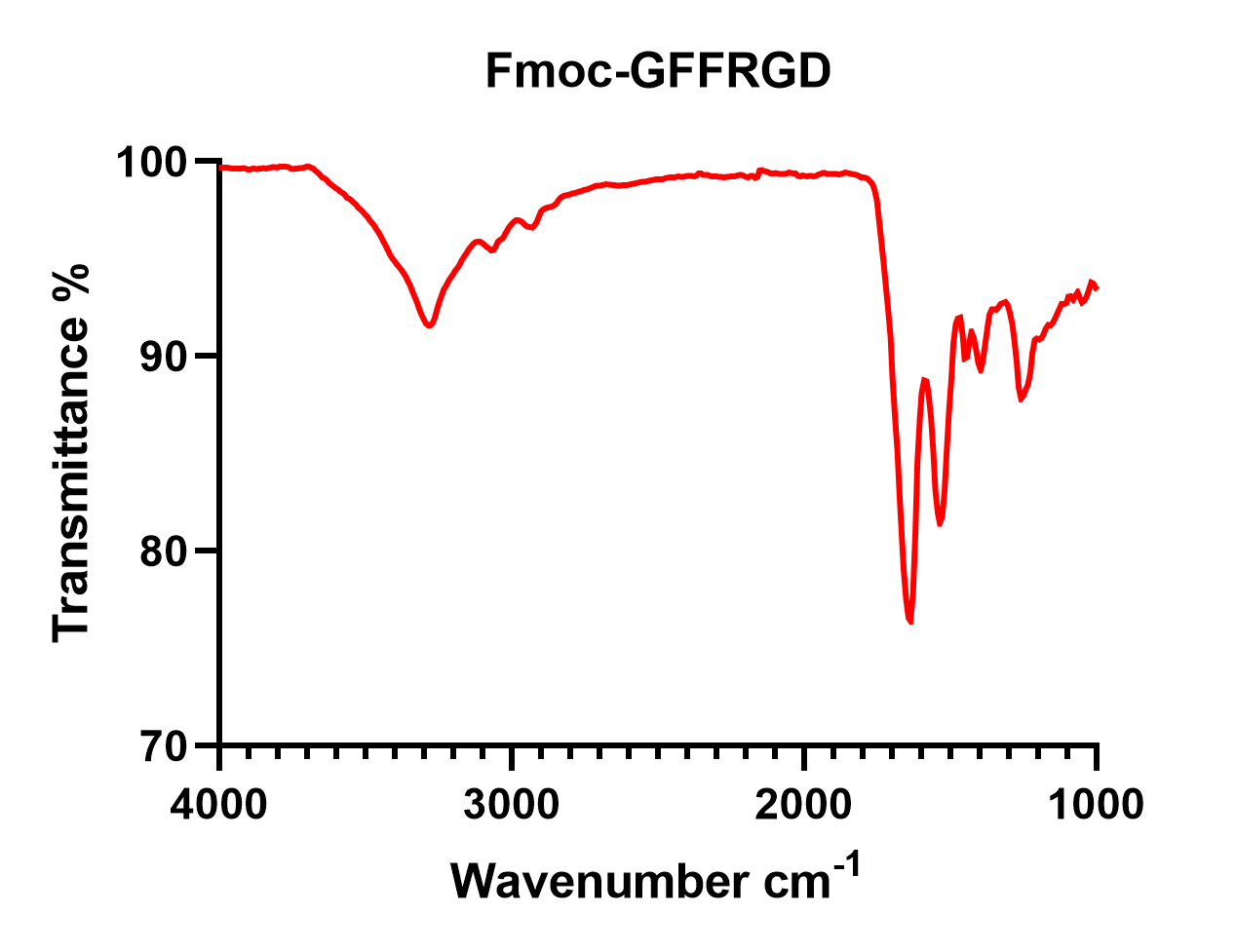


Figure S13. FTIR-ATR spectra of Fmoc-GFFRGD (solid).

# Supplementary Figures

c)

C)

b)

B)

a)

A)


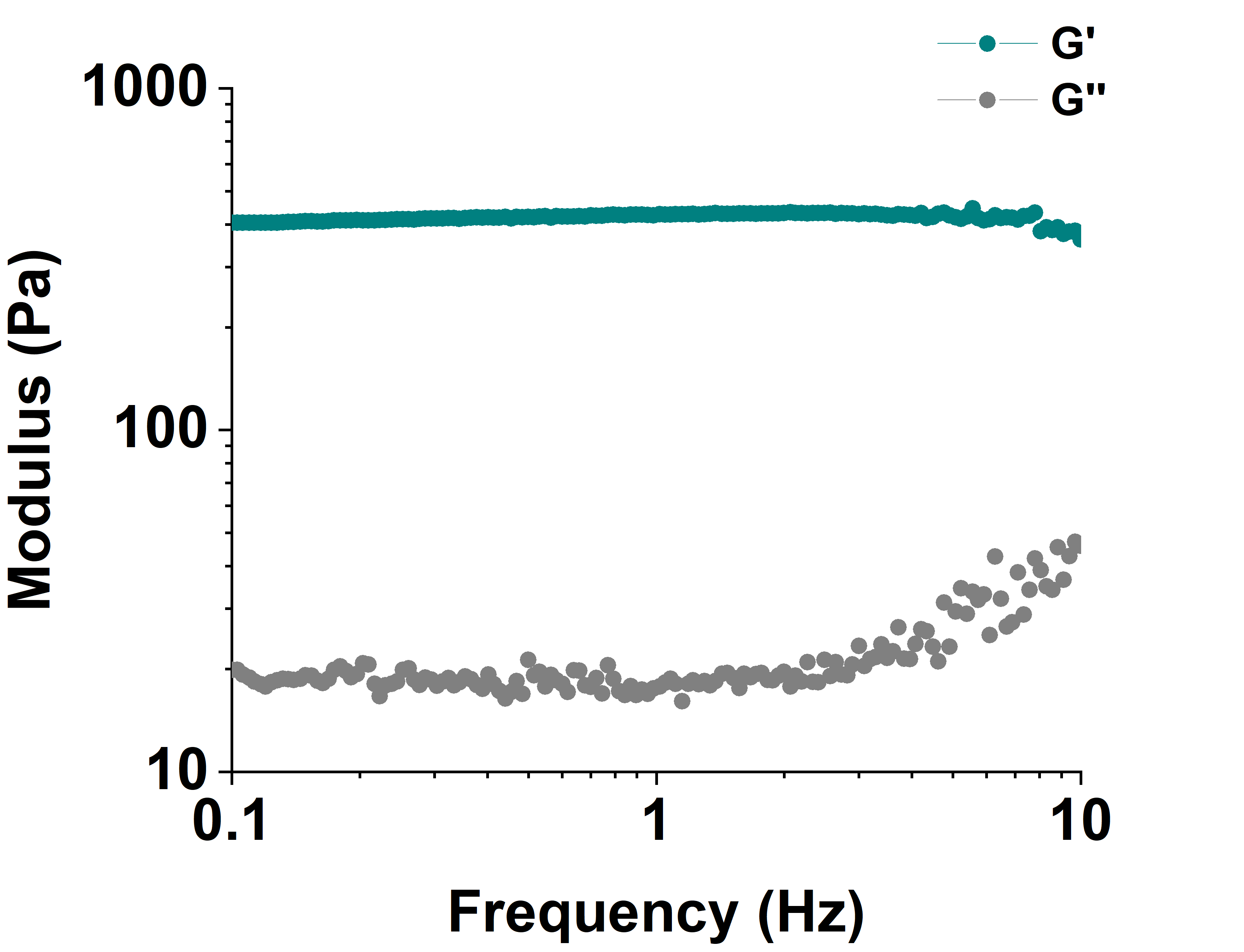

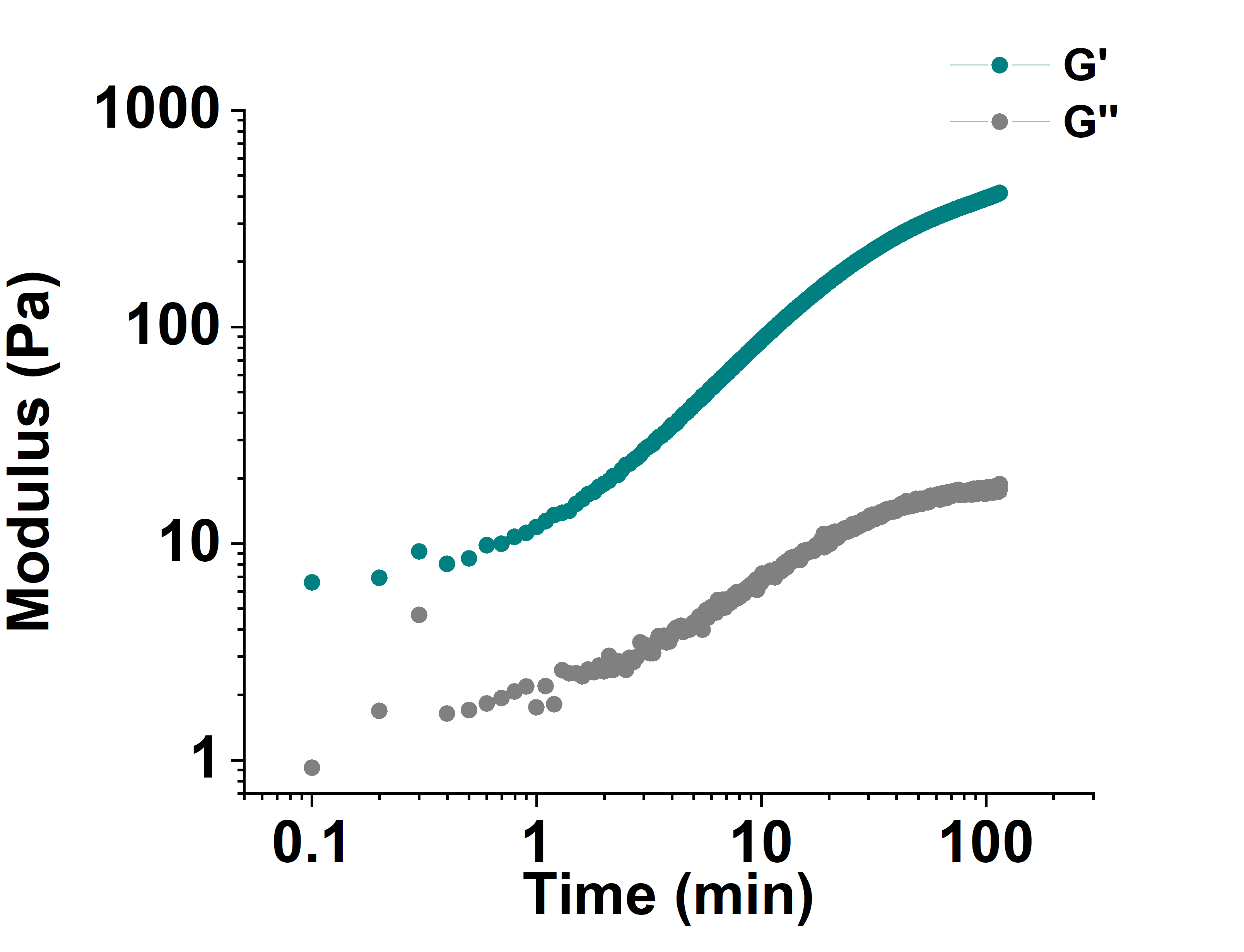

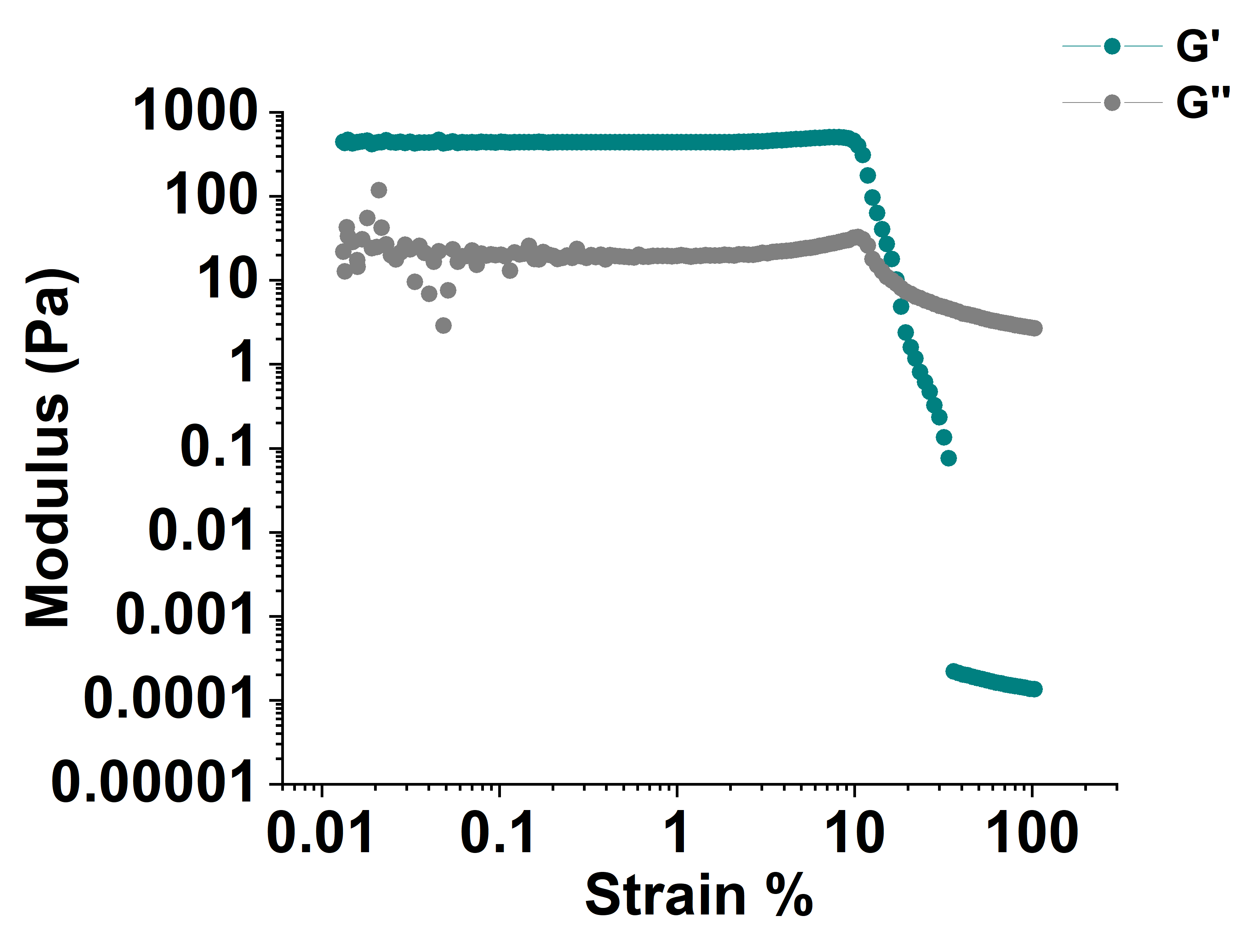

Figure S14. Rheological characterisation of scrFmoc-GFFRDG (0.4%, w/v in water:DMEM (1:1)) peptide hydrogel at 37 °C showing A) time sweep (at constant frequency of 1 Hz and strain of 0.2%), B) frequency sweep (logarithmic scale, strain = 0.2%) and C) strain sweep (frequency = 1 Hz). d) Tabulated data acquired from the three measurements.


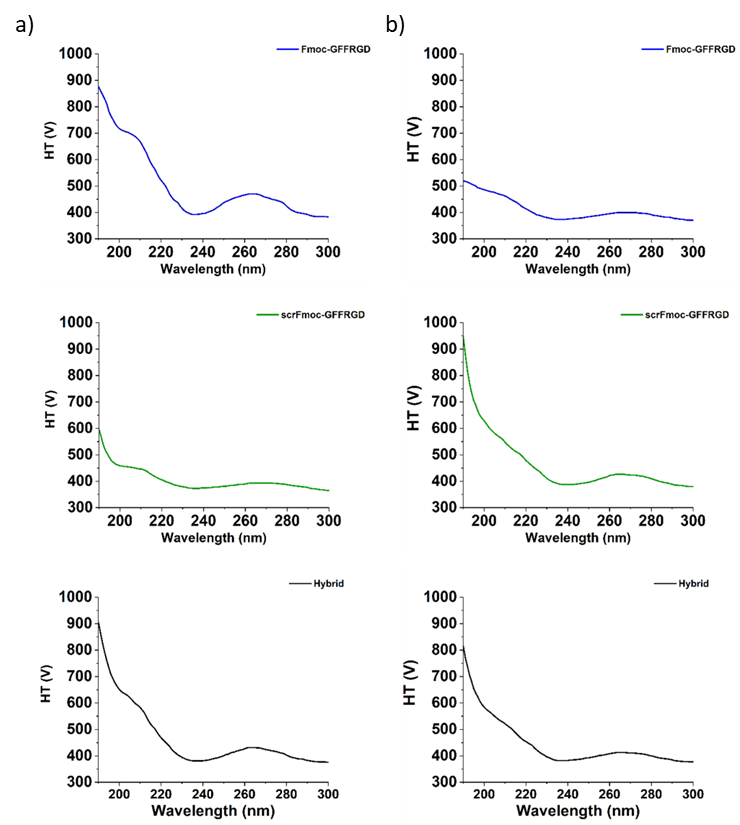


Figure S15. The circular dichroism HT signal corresponding to the CD spectra shown in Figure 2 of Fmoc-GFFRGD (blue line, 4.3 mM), scrFmoc-GFFRDG (green line, 0.5 mM) and hybrid (black line, 2.15 mM of each peptide; Fmoc-GFFRGD and scrFmoc-GFFRDG in total for both peptides is 4.3 mM) at 25 ° C prepared in: a) PBS buffer solution and b) hydrogel formed in water:DMEM (1:1, v/v).


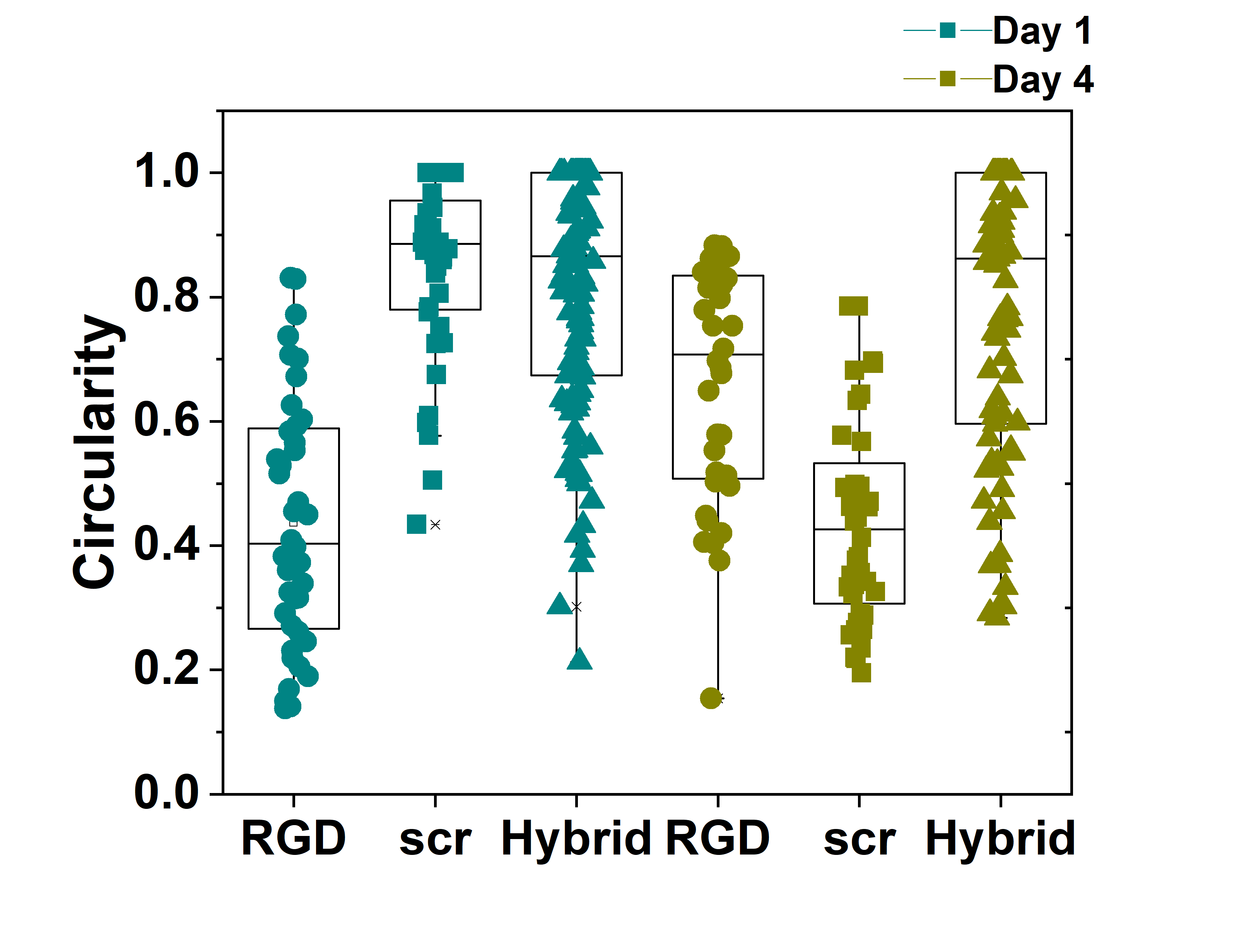


Figure S16. Circularity analysis (ImageJ) of Fmoc-GFFRGD and scrFmoc-GFFRDG. Value of 1.0 indicates a perfect circle and value close to 0.0 indicates an increasingly elongated cell. The circularity formula applied here is: 4π(area/perimeter2).

**References**

[1] C. P. Broedersz, K. E. Kasza, L. M. Jawerth, S. Münster, D. A. Weitz, F. C. MacKintosh, *Soft Matter* **2010**, *6*, 4120-4127.

[2] J. P. Wojciechowski, A. D. Martin, E. Y. Du, C. J. Garvey, R. E. Nordon, P. Thordarson, *Nanoscale* **2020**, *12*, 8262-8267
